# Ceria-Based Materials in Hydrogenation and Reforming Reactions for CO_2_ Valorization

**DOI:** 10.3389/fchem.2019.00028

**Published:** 2019-02-14

**Authors:** Marta Boaro, Sara Colussi, Alessandro Trovarelli

**Affiliations:** Dipartimento Politecnico, Università di Udine, Udine, Italy

**Keywords:** ceria based oxides, CeO_2_, CO_2_ methanation, CO_2_ valorization, methane dry reforming to syngas, gas to fuel technologies

## Abstract

Reducing greenhouse emissions is of vital importance to tackle the climate changes and to decrease the carbon footprint of modern societies. Today there are several technologies that can be applied for this goal and especially there is a growing interest in all the processes dedicated to manage CO_2_ emissions. CO_2_ can be captured, stored or reused as carbon source to produce chemicals and fuels through catalytic technologies. This study reviews the use of ceria based catalysts in some important CO_2_ valorization processes such as the methanation reaction and methane dry-reforming. We analyzed the state of the art with the aim of highlighting the distinctive role of ceria in these reactions. The presence of cerium based oxides generally allows to obtain a strong metal-support interaction with beneficial effects on the dispersion of active metal phases, on the selectivity and durability of the catalysts. Moreover, it introduces different functionalities such as redox and acid-base centers offering versatility of approaches in designing and engineering more powerful formulations for the catalytic valorization of CO_2_ to fuels.

## Introduction

For the first time since the Industrial Revolution the global CO_2_ atmospheric concentration have reached the threshold of 400 parts per million, increasing the average world temperature by 1.5°C within the next two-three decades (US, EPA, 2016; IPCC report, 2017). This poses a threat upon the environment and a great challenge for today's society, that must combine the drive toward a continued economic growth and ever-increasing demand of energy and chemicals with the need to preserve the environment for future generations. Among the main strategies which have been considered to reduce or minimize CO_2_ atmospheric emissions, one is its capture and storage (Araújo and de Medeiros, 2017). Carbon capture and storage (CCS) comprises separation of CO_2_ from industrial sources, compression and transportation to a geologic site for storage, or to enhanced oil recovery. Taking into account that the potential sequestration capability and the industrial use of CO_2_ are more than 150 times lower than its production, the CCS approach should be adopted only if we are able to develop technologies to convert efficiently CO_2_ into chemicals or fuels, in an alternative strategy, identified as CCSU, CO_2_ capture-storage-utilization (Cormos et al., 2018).

Therefore, in an envision of a free-carbon footprint circular economy, CO_2_ should substitute fossil carbon as feedstock to produce fuels and chemicals, while solar, wind, and geothermal sources should be employed for the production of electricity and H_2_ (Martens et al., 2017). Nowadays, there are many emerging technologies based on chemical catalysis, electrocatalysis, photocatalysis, and biocatalysis, but they are still not mature to make realistic the transition toward a mixed carbon-hydrogen economy (Kondratenko et al., 2013).

Since the fuel market is many times larger than the chemicals market, several efforts are directed to develop technologies to recycle back CO_2_ emissions to a synthetic transportable fuel. In this field, the highest readiness have been achieved by the technologies based on catalytic processes such as the reverse water gas shift (CO_2_+*H*_2_↔*CO*+*H*_2_O, RWGS), the methane dry reforming (CH_4_+*CO*_2_↔2*CO*+2*H*_2_, MDR), the methanation of CO_2_(*CO*_2_+4*H*_2_↔*CH*_4_+2*H*_2_O, CM), and CO_2_ conversion to oxygenates (Götz et al., 2016).

The methane dry reforming reaction is an endothermic process that occurs at high temperature (>800°C). This implies that the catalysts employed (mainly Ni, Co and related alloys) can sinter and coke due to CH_4_ cracking, moreover they can be deactivated by the presence of sulfurous compounds in the stream (Lavoie, 2014).

The direct hydrogenation of CO_2_ to methane is instead an exothermic process, thus thermodynamically favored at low temperature. A low operating temperature poses kinetic constrains which require efficient catalysts to be overcome. Various metals of the group VIIIB in the periodic table have been tested as catalysts for this reaction. Ru resulted one of the most active and selective as well as highly resistant to oxidizing atmosphere, however its high price limits its industrial application (Su et al., 2016). Nickel has been proved to be a valid alternative, especially for its low cost. The main disadvantage in using Ni is its high tendency to oxidize in the operating atmosphere, to poison in presence of sulfur gases and to volatilize forming nickel carbonyls, which are very toxic (Rönsch et al., 2016).

The reverse water gas shift reaction (RWGS) is a slightly endothermic process promoted mainly by copper based and supported ceria catalysts. Its main advantage is the formation of CO, which can be used as building block in other processes such the Fisher-Tropsch and the methanol syntheses. The production of methanol through the RWGS resulted competitive against that obtained by a direct hydrogenation of CO_2_, which is another possible reaction recently investigated to valorize CO_2._ Also in this case, the main issues are due to the need of a highly selective catalyst resistant to sintering and to poisoning due to coke and sulfur deposition (Daza and Kuhn, 2016).

A common feature of the above mentioned reactions is the simultaneous occurrence of several equilibrium reactions that may limit the selectivity of the process considered. Moreover, they require a highly active catalyst since CO_2_ is a very stable molecule. An important aspect in developing suitable catalysts to overcome the issues of these processes is the choice of an appropriate support. The support has a pivotal function in controlling the morphology and the oxidation state of the metal phase, its dispersion and, consequently, the activity and durability of the catalyst. Moreover, it can work as co-catalyst in the activation and dissociation of CO_2_. Among different supports CeO_2_ and mixed oxides based on ceria have proved to play a significant role in improving the catalytic performance for these processes.

The first catalytic application of ceria was as an oxygen storage component in automotive three-way catalysts but the use of ceria based oxides in catalysis is nowadays ubiquitous. Several articles give insight into the fundamentals of these materials and many recent reviews summarize their applications in catalysis (Trovarelli and Fornasiero, 2013; Montini et al., 2016; Xie S. et al., 2017; Devaiah et al., 2018). The superior catalytic capability of CeO_2_ are straightly linked to the reversible redox pair Ce^3+^/C^4+^ and to its surface acid basic properties (Trovarelli, 1996). Moreover, when nanostructured, or in its doped form, this oxide is characterized by a large number of surface defects, primarily oxygen vacancies (Małecka, 2016; Trovarelli and Llorca, 2017). The presence of these defects on the surface often alters dramatically the adsorption and subsequent reactions of various adsorbates on the support and on metal particles. Moreover it may contribute to generate very reactive metal-support interfaces (Mullinsn, 2015; Rodriguez et al., 2017). Shape and size of the ceria nanocrystals can be designed and controlled by different strategies of preparation and treatments to boost redox properties and enhance catalytic activity (Wu et al., 2016; Trovarelli and Llorca, 2017; Ma et al., 2018). With this basis the review analyzes the role of ceria and ceria doped oxides in the reforming processes for the valorization of CO_2_, highlighting advantages and disadvantages of their use. Emphasis is given to the peculiar role of ceria in the CO_2_ reforming processes mechanisms, in the perspective of designing cost-effective formulations based on ceria oxides that can boost the entry into the market of these technologies.

## CO_2_ Methanation Reaction

CO_2_ hydrogenation is in many cases the first step in the transformation of CO_2_ into valuable chemicals (Wang et al., 2011; Kondratenko et al., 2013). The reaction can lead to a series of products ranging from CO (via the reverse water gas shift) to alcohols and hydrocarbons, thus being of potential interest for several practical applications. CO_2_ hydrogenation to methane (1), known also as Sabatier reaction or CO_2_ methanation, represents a significant example of the conversion of waste (the CO_2_ emission) into energy (or energy carrier).

(1) $${\text{CO}}_{\text{2}}{\text{+4H}}_{\text{2}}\;↔\;{\text{CH}}_{\text{4}}{\text{+2H}}_{\text{2}}\text{O }(\Delta {\text{H}}_{\text{298K}}\;=\text{ -252}.\text{9 KJ/mol})$$

Among other implications, this reaction is a key step of the so called “Power-to-Gas” technology which nowadays is receiving great attention due to its potentialities in the renewable energy scenario (Götz et al., 2016; Rönsch et al., 2016; Li W. H. et al., 2018c). This process would allow an almost carbon neutral cycle by using hydrogen from renewables. The reduction of CO_2_ to methane proceeds via the transfer of eight electrons and presents strong kinetic limitations, thus requiring the presence of a suitable catalyst (Wei and Jinlong, 2011; Su et al., 2016). Catalysts for CO_2_ methanation are comprised by supported noble or transition metals, and there is a general agreement in the literature on the fact that the reaction mechanism is strongly influenced by the nature of the support (Su et al., 2016; Frontera et al., 2017).

### M-CeO_2_ Catalysts

Ceria-based catalysts have been found to possess higher activity and selectivity to methane with respect to other oxides for the methanation of carbon dioxide (Das and Deo, 2012; Tada et al., 2012; Atzori et al., 2017; Diez-Ramirez et al., 2017; Dreyer et al., 2017; Fukuhara et al., 2017; Martin et al., 2017; Li M. et al., 2018). The investigation of CO_2_ methanation on ceria-based materials dates back to the 90's, when firstly significant differences in this reaction between ceria and other supports were described (Trovarelli et al., 1991; de Leitenburg and Trovarelli, 1995). In particular, it was demonstrated that following a high temperature (500°C) reduction treatment the transient activity of a Rh/CeO_2_ catalyst was much higher compared to rhodium deposited on other oxides. This behavior was attributed to the presence of oxygen vacancies on the surface of reduced ceria, that were filled by the water formed during reaction thus explaining the transient nature of the high activity recorded for Rh/CeO_2_. Similarly, other noble metals supported on ceria showed a prominent transient behavior in CO_2_ methanation (de Leitenburg et al., 1997).

More recent studies revealed that indeed oxygen vacancies and ceria reducibility play an important role for CO_2_ hydrogenation to methane on metal-ceria systems, irrespective of the metal (Sharma et al., 2011, 2016; Tada et al., 2012; Upham et al., 2015; Wang et al., 2015; Wang F. et al., 2016; Zhou et al., 2016; Diez-Ramirez et al., 2017; Dreyer et al., 2017; Bian et al., in press). Wang et al. compared the activity of Ru supported on ceria nanocubes (NC), nanorods (NR), and nanopolyhedra exposing different facets for CO_2_ methanation (Wang et al., 2015). Their results clearly demonstrate that the most active catalyst, i.e., Ru on CeO_2_ nanocubes, is the one that shows the highest concentration of oxygen vacancies which serve as active sites for CO_2_ activation. In a similar study carried out for Ni supported on ceria nanocubes and nanorods, instead, the highest activity was recorded over Ni/CeO_2_-NR, which presented a higher number of Ce^3+^ sites (Bian et al., in press). The apparent contradiction is due to the fact that in the first study the most defective structure was that of ceria nanocubes, whereas in the second it was that of ceria nanorods. This is likely linked to the variability of the defective structure of ceria nanorods, for which growth direction of crystal planes, exposed facets, and presence of defects may depend on synthesis approach (Trovarelli and Llorca, 2017).

#### Mechanism of Reaction

**Figure 1:**
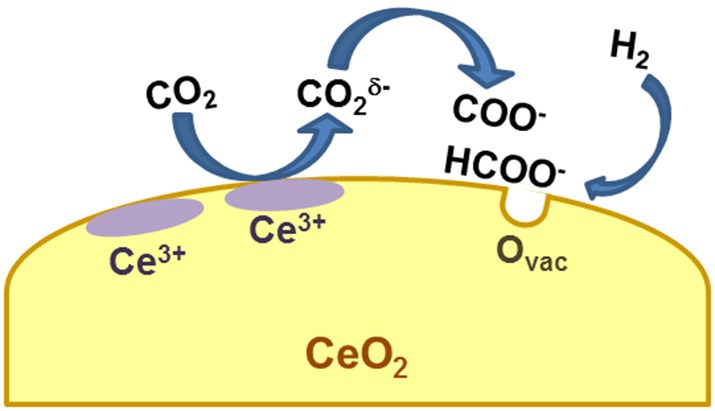
Proposed mechanism for CO_2_ activation on ceria surface.

The coupling of advanced characterization techniques with *in situ* and theoretical studies allowed to uncover the effect of CeO_2_ redox properties: the reduced and basic ceria surface favors the adsorption of CO_2_ and its decomposition, while the presence of oxygen vacancies catalyzes the formation and dissociation of reaction intermediates (Figure 1).

Regarding CO_2_ adsorption/activation, it is reported that this happens to a significant extent only on reduced ceria or in presence of H_2_, indicating that the presence/formation of a vacancy is a necessary prerequisite (Sharma et al., 2016). Beside the first CO_2_ chemisorption step, the mechanisms proposed in the literature for the hydrogenation of carbon dioxide to methane on ceria-based systems, obtained from *in situ* or *operando* measurements, follow two major routes: the formate and/or the carbonate route. Wang et al. couple steady-state isotope transient kinetic analysis (SSITKA)-type *in situ* DRIFT infrared spectroscopy with *operando* XANES and Raman spectroscopy to observe the evolution of intermediates and surface species during CO_2_ hydrogenation over Ru/CeO_2_ nanocubes (Wang F. et al., 2016). According to their results recorded from 25 to 400°C, CO_2_ converts to carboxylate species ${\text{CO}}_{2}^{\text{δ}-}$ by interacting with surface Ce^3+^ and this step is followed by the reaction of ${\text{CO}}_{2}^{\text{δ}-}$ with surface hydroxyls to produce formate (HCOO^−^) species. Formates dissociation to methanol then takes place at around 300°C with subsequent hydrogenation of methanol to methane. The presence of reduced ceria (Ce^3+^) is necessary to initiate the reaction, and based on the observation reported in this work it changes drastically the picture with respect to Ru on alumina support, on which CO_2_ chemisorption gives rise to the formation of bicarbonates. In agreement with this work, the evolution of formates on Ru/CeO_2_ and bicarbonates on Ru/Al_2_O_3_ has been observed also by Dreyer et al. (2017). A different situation is described by Sharma et al., who observe the formation of carbonate species on a Ru-substituted ceria catalyst by *in situ* DRIFT (Sharma et al., 2016). The discrepancy might be due to the fact that in this last example the metal is substituted in the ceria lattice, giving rise to a different configuration of the metal-ceria entity. Nevertheless, also in this case the authors postulate the need of a reduced ceria surface and/or the presence of oxygen vacancies to activate the CO_2_. Also in the case of Ni-based catalysts there are some apparent contradictions. The carbonate route has been described for example by Zhou and coworkers for Ni/CeO_2_ catalysts, prompted again by the formation of oxygen vacancies (Zhou et al., 2016). The formate route is proposed instead by Bian and coworkers, who recently studied the methanation activity of Ni supported on ceria nanorods (Bian et al., in press). They also link the evolution of formates to the presence of reduced Ce^3+^ sites. Other authors have observed the coexistence of the two reaction intermediates (formate and carbonate species) on Ni/CeO_2_ and Rh/CeO_2_ catalysts (Konishcheva et al., 2016; Martin et al., 2017), thus leading to the conclusion that both routes can take place, jointly or separately, likely depending on reaction conditions. A theoretical work by Lu and coworkers describes the occurrence of different paths for CO_2_ reduction on ceria (111), demonstrating that indeed the nature of the surface (defective or stoichiometric) and of the reaction environment is crucial for the establishment of one or another reaction route (Lu et al., 2015). Despite the different mechanisms reported, there is a general agreement on the role of ceria which is always that of adsorbing and activating CO_2_ via a redox mechanism involving Ce^3+^ and/or oxygen vacancies. A recent study by Li et al. on an Ir/CeO_2_ catalyst sheds some light on another important element, i.e., the effect of ceria in tuning the chemical properties of the metal (Li et al., 2017), another issue that might explain the different mechanisms reported by different research groups for the same metal. In this work it is reported that the selectivity toward methane (or carbon monoxide) strongly depends upon Ir oxidation state, which in turn is determined by the capability of ceria to transfer oxygen atoms to Ir nanoparticles due to a strong metal support interaction. It is clear then that different preparation routes and/or different metal loadings can change the way ceria interacts with the metal itself, thus affecting the reaction mechanism.

### Ceria as Promoter

Ceria has not been studied only as a support for methanation reaction but also as a dopant on other oxides, with the aim of improving the overall catalytic activity. The addition of CeO_2_ has been shown to enhance the reducibility of the catalysts (Liu et al., 2012; Tada et al., 2014; Abate et al., 2016; Toemen et al., 2016), to increase metal dispersion (Bian et al., 2015; Nie et al., 2017), to improve catalyst stability (Liu et al., 2012; Toemen et al., 2016; Nie et al., 2017), and to promote CO_2_ adsorption by modifying the basicity of the supports (Westermann et al., 2017). In particular, in the case of Ni-Al_2_O_3_ catalyst it was found that a little amount of CeO_2_ (2–3 wt%) was enough to enhance not only the catalytic performances but also the stability during long time operation, while a further increase in ceria content resulted in a slight activity loss (Liu et al., 2012; Nie et al., 2017). Nevertheless, from a literature survey it is not straightforward to determine the optimum ceria loading for Ni-alumina catalysts, since a positive effect was reported also for much higher (60%) ceria content (Bian et al., 2015).

### M-CeO_2_-ZrO_2_ Catalysts

When ceria is coupled with zirconia all the above mentioned aspects are emphasized, and where a comparison with pure ceria is reported the mixed oxide shows a better catalytic activity (Razzaq et al., 2013; Zhu et al., 2013). The enhanced performances of Ce-Zr based catalysts with respect to pure ceria can be ascribed to the higher number of defects, which are known to form upon addition of Zr^4+^ ions into the lattice of ceria, and the subsequent increase in oxygen mobility. Most of the papers dealing with ceria-zirconia as support or promoter for CO_2_ methanation involve nickel (Aldana et al., 2013; Cai et al., 2013; Pan Q. et al., 2014; Nizio et al., 2016; Ashok et al., 2017; Le et al., 2017) or Ni-based bimetallic formulations (Ocampo et al., 2011; Razzaq et al., 2013; Zhu et al., 2013; Pastor-Pérez et al., 2018; Shang et al., 2018) as the active phase. In general, these studies highlight the high reducibility of ceria-zirconia based catalysts and their remarkable stability during time on stream operation, also when CeO_2_-ZrO_2_ is used as a dopant (Le et al., 2017). The former aspect is clearly due to the well-known redox properties and oxygen storage capacity (OSC) of Ce-Zr solid solutions, whereas the latter appears to be related to the interaction between the metal and the support which can avoid or limit particle sintering. A strong metal-support interaction has a positive effect on catalytic activity both in transient (i.e., during temperature ramping) and in steady state (isothermal) conditions, as shown by comparing the behavior of Ni-CeZr prepared by different methods giving rise to different degrees of interaction between Ni and ceria-zirconia (Aldana et al., 2013; Ashok et al., 2017). The higher methanation activity and stability during time-on-stream operation has been attributed to Ni species strongly interacting with the support and to the higher mobility of surface oxygen/hydroxyl species. When compared with other supports, ceria-zirconia is found indeed to improve metal-support interaction with beneficial effect on catalytic performance (Razzaq et al., 2013). Moreover, an opportune tailoring of the synthesis procedure, for example by incorporation of Ni ions into Ce-Zr lattice by impregnation or ammonia evaporation, can lead to samples with higher metal dispersion, oxygen vacancies and reduced ceria sites (Pan Q. S. et al., 2014; Ashok et al., 2017). Also the addition of another metal such as Ru (Shang et al., 2018) or Co (Zhu et al., 2013; Pastor-Pérez et al., 2018) can promote the activity and stability of Ni-CeZr formulations, in particular by stabilizing Ni dispersion, reducing carbon deposits and increasing the amount of basic sites.

Different aspects should be taken into account if looking for the optimum ceria-zirconia composition, which seems to depend upon several factors. For example the role of surface area is not univocal comparing various Ce/Zr molar or mass ratios, and this is likely due to different preparation methods, experimental conditions, Ni loading etc. For some authors the best results are actually obtained on samples with the highest surface area (Cai et al., 2013; Zhu et al., 2013), while in other studies this is not the case. The ratio between Ce and Zr has been found to influence the amount and distribution of Ni^2+^ and Ni^0^ species, the best composition being thus the one that guarantees the optimum of these active centers (Ocampo et al., 2011). Ce/Zr ratio has been reported to affect also metal dispersion and the amount and strength of basic sites (Nizio et al., 2016). Regarding the mechanism of CO_2_ hydrogenation to methane on ceria-zirconia based catalysts, the carbonate/formate route seems confirmed by *in situ* DRIFT experiments, which also exclude the formation of CO as a reaction intermediate (Aldana et al., 2013; Pan Q. et al., 2014; Ashok et al., 2017).

Performances of the catalysts above discussed are summarized in Table 1.

**Table 1:** Survey of CO_2_ methanation ceria based catalysts considered in this review.

**Catalyst**	**Preparation method**	**Metal (nm)**	**T°C**	**Reaction conditions**	**XCO_**2**_ (%)**	**YCH_**4**_ (%)**	**CH_**4**_/CO**	**Stability test^c^**	**References**
**CO**_**2**_ **methanation on M/CeO**_**2**_ **catalysts (M = Ru, Co, Ir, Ni)**									
Ce_0.95_Ru_0.05_O_2_	Solution combustion synthesis	nd	450	H_2_/CO_2_ = 4 dil., 15 mL/min, mass_cat_ 0.02 g	55	nd	99	Stable for 16 h at 325°C	Sharma et al., 2011
Ce_0.95_Ru_0.05_O_2−δ_	Solution combustion synthesis	nd	350	H_2_/CO_2_ = 4 dil., 10 mL/min, mass_cat_ 0.025 g	40	40	99	nd	Upham et al., 2015
3%Ru/CeO_2_-NC	CeO_2_ NC hydrothermal Ni DP	3.7	150	H_2_/CO_2_ = 4 dil., 40 mL/min, mass_cat_ 1 g	4.85*10^−8^ mol/${\text{g}}_{\text{cat}}^{*}$s^b^	nd	99	nd	Wang et al., 2015
3%Ru/CeO_2_	Flame spray pyrolysis	1.6	300	H_2_/CO_2_~5, GHSV = 7,640 h^−1^	83	nd	99	nd	Dreyer et al., 2017
5–20%Co/CeO_2_	W-IMP	nd	260	H_2_/CO_2_ = 25, 52 mL/min, mass_cat_ 0.06 g	0.8–6.7	0.3–6.5	nd	nd	Das and Deo, 2012
42.3%Co/CeO_2_	W-IMP	27.4^a^	300	H_2_/CO_2_ = 9, 75 mL/min, mass_cat_ 0.6 g	96	96	100	Stable for 24 h	Diez-Ramirez et al., 2017
0.7–20%Ir/CeO_2_	Adsorption-precipitation	1–2.2^d^	300	H_2_/CO_2_ = 4 dil., 20 mL/min, mass_cat_ 0.1 g	2.9–8.8	nd	<1–88	nd	Li et al., 2017
10%Ni/CeO_2_	W-IMP	17^a^	250 350	H_2_/CO_2_ = 5, GHSV = 10,000 h^−1^	28 95	nd	100	nd	Tada et al., 2012
7.4%Ni/CeO_2_	W-IMP	10	250	H_2_/CO_2_ = 65 dil., GHSV = 29 L·gcat^−1^h^−1^	100	nd	nd	Stable for 20 h	Konishcheva et al., 2016
10%Ni/CeO_2_	Excess Imp.	nd	340	H_2_/CO_2_ = 4.6 dil., GHSV = 22,000 mL·gcat^−1^h^−1^	98.1	nd	100	7% in 10 h	Zhou et al., 2016
5–35%Ni/CeO_2_-HT 5–35%Ni/CeO_2_-IWI	Hard template W-IMP Ni on CeO_2_ HT	nd-16 nd-23	300	H_2_/CO_2_ = 4.4 dil., GHSV = 72,000 mL·gcat^−1^h^−1^	37–76 48–57	nd	93–99 97–98	Stable for 30 h (15%Ni/CeO_2_ IWI and HT)	Atzori et al., 2017
10%Ni/CeO_2_	Impregnation	20	200 300	H_2_/CO_2_ = 4 dil., 70 mL/min, mass_cat_ 0.3 g	20 84	nd	100	Stable for 125 h at 350°C	Fukuhara et al., 2017
3%Rh/CeO_2_ 3%Ni/CeO_2_	W-IMP	<3	350	H_2_/CO_2_ = 5 dil., 2 L/min, GHSV = 60,000 h^−1^	44 40	~98 80	nd	nd	Martin et al., 2017
48.9%Ni/CeO_2_ ST 53.8%Ni/CeO_2_ IWI	Soft template W-IMP Ni on CeO_2_ ST	4 21	300	H_2_/CO_2_ = 4.3 dil., GHSV = 72,000 mL·gcat^−1^h^−1^	87 83	nd	>99.5	Stable for 6 h	Atzori et al., 2018
5%Ni/CeO_2_-NR	Impregnation	nd	200 250	H_2_/CO_2_ = 4 dil., GHSV = 24 L·gcat^−1^h^−1^	2.5 24	nd	~97 ~100	nd	Bian et al., in press
5%Ni/CeO_2_	W-IMP	8.7	250	H_2_/CO_2_ = 4 dil., GHSV = 16,000 h^−1^	TOF 271 h^−1^	nd	100	~7% in 50 h at 66,000 h^−1^	Li M. et al., 2018
**CO**_**2**_ **methanation on M/Ce/OX catalysts (M = Ru, Ni, OX = support oxide)**									
2%Ru/30%CeO_2_/Al_2_O_3_	W-IMP	16	350	H_2_/CO_2_ = 4 dil., GHSV = 10,000 h^−1^, mass_cat_ 0.3 g	~95	nd	~100	nd	Tada et al., 2014
5%Ru/65%Ce/30%Mn/Al_2_O_3_	W-IMP	nd	200	H_2_/CO_2_ = 4 dil., GHSV = 636 mL·gcat^−1^h^−1^	97.7	91.3	nd	Stable for 20 h at 300°C	Toemen et al., 2016
15%Ni/2%CeO_2_/Al_2_O_3_	Co-impregnation	nd	350	H_2_/CO_2_ = 4, GHSV = 15,000 mL·gcat^−1^h^−1^	85	nd	100	Stable for 120 h	Liu et al., 2012
15%Ni/60%CeO_2_/Al_2_O_3_	Dielectric barrier discharge plasma	5.3	250	H_2_/CO_2_ = 4, GHSV = 30,000 mL·gcat^−1^h^−1^	63	nd	~96	nd	Bian et al., 2015
20%Ni/15%CeZrTi/Al_2_O${}_{3}^{\text{e}}$	Impregnation precipitation	17	250	H_2_/CO_2_ = 4 dil., GHSV = 20,000 h^−1^, mass_cat_ 0.6 g	44.4	43.4	~99	Stable for ~7 h at 300°C	Abate et al., 2016
20%Ni/3%CeO_2_/Al_2_O_3_	Co-impregnation	6.5	300	H_2_/CO_2_ = 4, GHSV = 3,000 mL·gcat^−1^h^−1^	~96	nd	99.5	Stable for 150 h	Nie et al., 2017
5%Ni/15%CeO_2_/USY zeolite	W-IMP	nd	350	H_2_/CO_2_ = 4 dil., GHSV = 43,000 h^−1^, mass_cat_ 0.6 g	~30	nd	~90	nd	Westermann et al., 2017
**CO**_**2**_ **methanation on M/CeZr catalysts (M = Ni and bimetallic Ni-M compositions)**									
5%Ni/Ce_0.5_Zr_0.5_O_2_	Pseudo sol-gel	21	350	H_2_/CO_2_ = 4 dil., GHSV = 43,000 h^−1^, mass_cat_ 0.15 g	~80	nd	99	12% in 90h	Aldana et al., 2013
5%Ni/Ce_0.72_Zr_0.28_O_2_	CeZr hydration Ni W-IMP	nd	350	H_2_/CO_2_ = 4 dil., GHSV = 35,400 h^−1^, mass_cat_ 0.5 g	68.9	58.2	90.2	10% in 60h at 390°C	Cai et al., 2013
7%Ni/Ce_0.5_Zr_0.5_O_2_	CeZr homogeneous precipitation Ni W-IMP	nd	340	56% CH_4_, 33%H_2_O, 9% H_2_, 2%CO_2_, p = 3 MPa, GHSV = 20,000 h^−1^	~70	nd	nd	nd	Pan Q. et al., 2014
10%Ni/Ce_0.5_Zr_0.5_O_2_	CeZr homogeneous precipitation Ni W-IMP	14.4	340	56% CH_4_, 33%H_2_O, 9% H_2_, 2%CO_2_, p = 3 MPa, mass_cat_ 2 g GHSV = 20,000 h^−1^	73	nd	100	nd	Pan Q. S. et al., 2014
15%Ni/Ce_0.58_Zr_0.42_O_2_	W-IMP	18.7	300	H_2_/CO_2_ = 4, GHSV = 50,000 h^−1^	80	nd	100	nd	Nizio et al., 2016
10%Ni/CeZrO_2_, Ce/Zr = 1.35	Ammonia evaporation	4.6	275	H_2_/CO_2_ = 4 dil., mass_cat_ 0.15 g GHSV = 20,000 mL·gcat^−1^h^−1^	55	nd	99.8	stable for 70h	Ashok et al., 2017
7% Ni/Ce_0.2_Zr_0.8_O_2_/AC^f^	CeZr hydrothermal CeZr/AC suspension Ni W-IMP	17.4	350	H_2_/CO_2_ = 4 dil., mass_cat_ 0.3 g GHSV = 4,000 mL·gcat^−1^h^−1^	85	nd	100	nd	Le et al., 2017
5%Ni/Ce_0.6_Zr_0.4_O_2_ 5%Ni/0.5%Rh/Ce_0.8_Zr_0.2_O_2_	Pseudo sol-gel	20.8 16.1	350	H_2_/CO_2_ = 4 dil., GHSV = 43,000 h^−1^	80 78	nd	99 99	14% in 150h16% in 150h	Ocampo et al., 2011
15%Ni/5%Co/Ce_0.5_Zr_0.5_O_2_	CP	nd	400	7.04% CO, 3.05% CO_2_, 4.05% N_2_, 27.23%CH_4_, 58.63% H_2_ GHSV = 5,000 h^−1^	90	nd	100	nd	Razzaq et al., 2013
15%Ni/5%Co/Ce_0.25_Zr_0.75_O_2_	CP in presence of PEG-6000	nd	280	H_2_/CO_2_~4 dil., GHSV = 10,000 h^−1^	85	nd	98	8% in 120h	Zhu et al., 2013
15%Ni/3%Co/CeZrO_2_ Ce/Zr = 1.5	CeZr CP Ni, Co IMP	11	300	H_2_/CO_2_ = 4 dil., GHSV = 12,500 mL·gcat^−1^h^−1^	83	nd	93	10% in 50h GHSV = 14,000 mL·gcat^−1^h^−1^	Pastor-Pérez et al., 2018
30%Ni/3%Ru/Ce_0.9_Zr_0.1_O_2_	One-pot hydrolysis	11.7^a^	230	H_2_/CO_2_ = 4, GHSV = 2,400 mL·gcat^−1^h^−1^	98.2	nd	100	stable for 300hGHSV = 4,800 mL·gcat^−1^h^−1^	Shang et al., 2018

a *After reduction*.

b *Reaction rate*.

c *Decrease in % of methane production*.

d *After reaction*.

e 15%CeO_2_-15%ZrO_2_-15%TiO_2_/55%Al_2_O_3._

f *AC, Activated Carbon*.

## CO_2_ Hydrogenation to Value Added Chemicals Other than Methane

The products of CO_2_ hydrogenation different from methane are usually divided into three main groups, depending on the reaction pathway, i.e., methanol synthesis, reverse water gas shift (RWGS) and CO_2_ Fischer-Tropsch (CO_2_-FT), the latter two being intimately linked with each other (Porosoff et al., 2016). The fundamental properties of ceria and ceria-zirconia for CO_2_ methanation play a crucial role also in these CO_2_ hydrogenation reactions, for which the degree of CO_2_ reduction by hydrogen, beside other factors such as temperature and H_2_/CO_2_ ratio, is generally controlled by the nature of the metal active phase. Pt/CeO_2_ for example is highly selective toward CO with respect to methane as well as Cu/CeO_2_, whereas Co and Ni-based catalysts are selective toward methane (Porosoff and Chen, 2013; Dai et al., 2017).

### Methanol Synthesis

#### M-CeO_2_ Catalysts

Metal-ceria interaction, and particularly metal-ceria interface, is found to be a key parameter for the hydrogenation of CO_2_ to methanol (Rodriguez et al., 2015; Kattel et al., 2017). This reaction is widely investigated as methanol is a valuable raw material and an intermediate for the synthesis of other chemicals (Alvarez et al., 2017). Recent studies on inverse ceria-copper catalysts reveal the importance of the superficial interaction between the two components (Graciani et al., 2014; Senanayake et al., 2016; Rodriguez et al., 2017). Inverse oxide/metal configurations are regarded in general as more active than traditional metal/oxide systems and can provide stronger bonding at the interface due to perturbations of the electronic properties of the oxide. This is indeed the case of CeO_x_/Cu(111), whose superior activity for methanol production is attributed to the abundance of Ce^3+^ sites that can stabilize ${\text{CO}}_{2}^{\text{δ}-}$ intermediates. The synergistic effect between the metal and CeO_2_ has been reported also on conventional systems in which the metal is deposited onto the ceria surface, and again the enhanced activity for CO_2_ hydrogenation has been attributed to the increased reduction of ceria promoted by metal-support interaction and the subsequent formation of oxygen vacancies (Tsubaki and Fujimoto, 2003; Choi et al., 2017; Vourros et al., 2017; Malik et al., 2018). The relevance of this interaction is nicely demonstrated by Vourros et al., who compares the activity of gold nanoparticles supported on different oxides. From this work it appears clearly that when Au is deposited on ceria the activity for CO_2_ hydrogenation to methanol depends almost uniquely on the metal-support interplay, ceria being inert for the reaction as well as gold supported on alumina (Vourros et al., 2017). Also, in the earlier paper of Tsubaki and Fujimoto it is reported that depending on the degree of sample reduction (at low or high temperature), the hydrogenation of carbon dioxide is selective toward methane (T_red_ = 200–400°C) or methanol (T_red_ = 500–550°C) on Pd/CeO_2_ catalyst. This is attributed to a weaker or stronger bonding of CO on catalyst surface, which in turn depends on the reciprocal arrangement of Pd and ceria, that leads to CH_4_ (in the case of low temperature reduction) or to CH_x_O intermediates (for high temperature reduction), respectively (Tsubaki and Fujimoto, 2003). The synergy between metal and support can be tailored also by choosing opportune ceria facets, as demonstrated by carrying out CO_2_ hydrogenation on copper supported on ceria with different morphologies (Ouyang et al., 2017). In this case it was found that the best catalytic activity is given by Cu on ceria nanorods exposing the (100) and (110) faces, which guarantee the strongest Cu-CeO_2_ interaction.

#### CeO_2_ as Promoter

Ceria has been successfully applied also as a promoter to conventional Cu/Zn/Al and other oxide based catalysts for methanol synthesis from CO_2_ and H_2_ (Richard and Fan, 2018). In particular, it is reported that for Cu-based catalysts the addition of ceria can increase the number of basic sites and the dispersion of the supported metal (Gao et al., 2013; Zhan et al., 2014; Shi et al., 2018), and can change the electronic environment of copper promoting the metal-ceria interaction (Wang et al., 2002; Bonura et al., 2011; Ban et al., 2014).

#### Mechanism of Reaction

**Figure 2:**
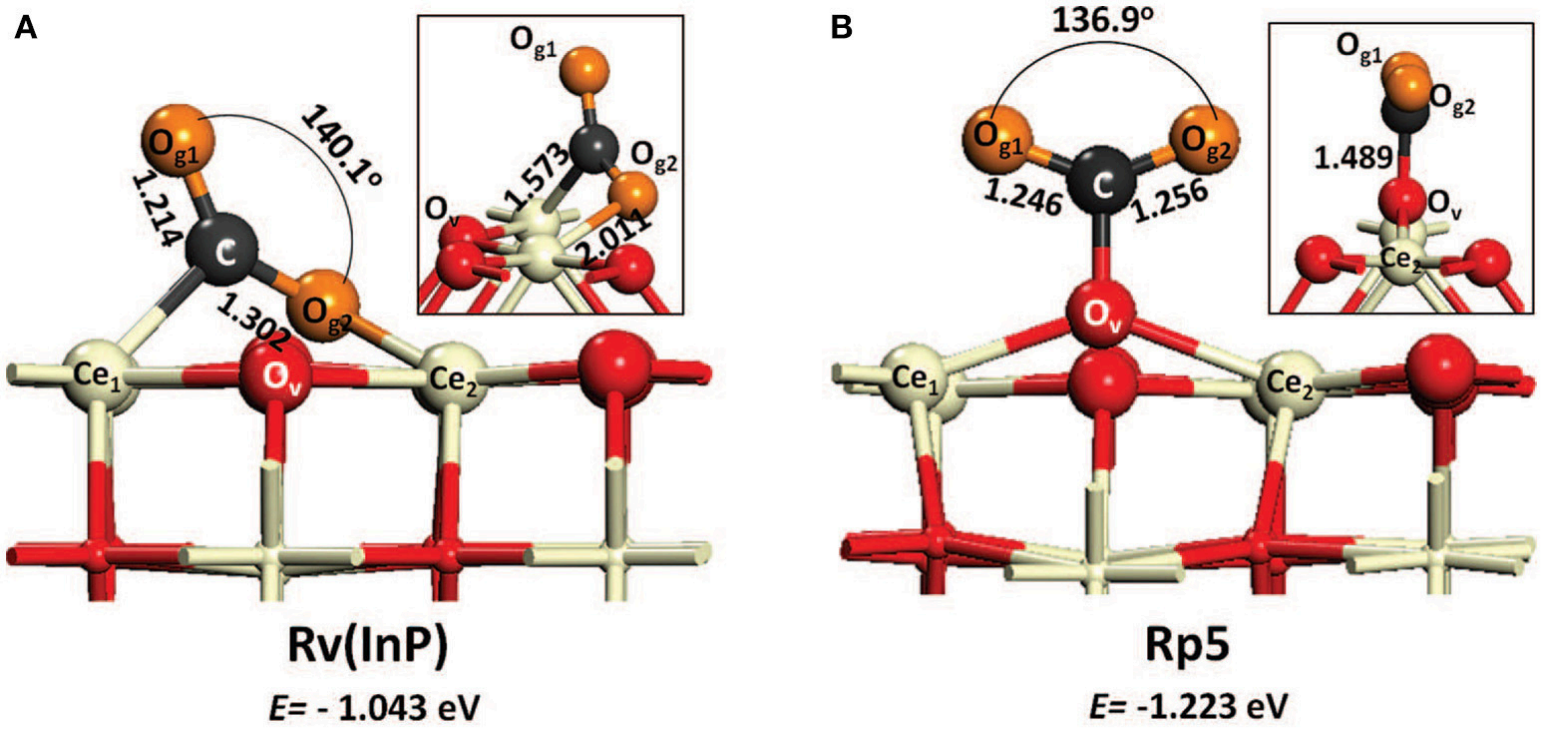
Two CO_2_ adsorption configurations on reduced ceria (110) with in-plane **(A)** vacancy [Rv(InP)] and **(B)** split vacancy (Rp5). The surface oxygen nearest the vacancy is labeled as O_v_. Both structural parameters and energies of adsorption are depicted (from Cheng et al., 2013, reproduced with permission of AIP Publishing).

The mechanism of CO_2_ hydrogenation to methanol described in the literature involves the formation of carboxylate species and CH_x_O intermediates excluding a role of formates, due to their too high stability (Graciani et al., 2014; Rodriguez et al., 2015). Formates, which were instead the main intermediates for CO_2_ methanation (see section CO_2_ Methanation Reaction), are indeed observed also in this case but they are identified as spectators (Senanayake et al., 2016). This mechanism and relative observations are in agreement with the theoretical work by Kumari et al. who investigated CO_2_ hydrogenation on CeO_2_(110) surface and reported a tight bond of formate intermediates on ceria, thus making their participation in the reaction unlikely (Kumari et al., 2015). On the other side, a recent experimental paper by Malik et al. describe the formate route as the most probable and their results agree with the microkinetic analysis performed by Cheng and Lo again on CeO_2_(110) surface (Cheng and Lo, 2016). It is not easy to understand the reasons of these apparent contradictory results. For example Cheng and Lo suggest that at moderately high H_2_/CO_2_ ratio the formate route is favorable, so one might argue that the value of 9 for H_2_/CO_2_ ratio used in the work by Graciani et al. is too high, or that the different catalyst configuration (inverse CeO_2_/Cu catalyst for Graciani, conventional PdZn/CeO_2_ for Malik) makes the difference. An interesting explanation, which could account in general for the discrepancies reported in the literature for CO_2_ hydrogenation routes and intermediates formed on ceria surfaces, is that inferred from the theoretical work by Cheng et al. It is observed that depending on the type of vacancy (in site or split) the binding of CO_2_ on ceria surface is different (Figure 2). This leads to different paths of CO_2_ activation (Cheng et al., 2013). Whichever the explanation, theory and experiments agree in highlighting the role of the reduced ceria surface, similarly to what reported for CO_2_ methanation, with oxygen vacancies responsible of charge redistribution and consequent CO_2_ activation.

### Reverse Water Gas Shift Reaction

The reduction of CO_2_ to CO by the reverse water gas shift (2) is another fundamental reaction for the production of valuable chemicals, for example as the first step of the Fischer-Tropsch synthesis (Wang et al., 2011; Centi et al., 2013; Daza and Kuhn, 2016; Yang et al., 2018).

(2) $${\text{CO}}_{\text{2}}\text{ + }{\text{H}}_{\text{2}}\;↔\text{ CO + }{\text{H}}_{\text{2}}\text{O }(\Delta {\text{H}}_{\text{298K}}\;=\text{ 41}.\text{2 KJ/mol})$$

From a thermodynamics point of view, the reverse water gas shift (RWGS) is an equilibrium limited endothermic reaction favored at high temperature. It is a very promising route for the valorization of CO_2_ because of the technical feasibility and readiness of the process. Several authors investigated this reaction, but there are comparatively few studies specifically devoted to RWGS on ceria-based materials, whereas much more work involves it indirectly because it takes place to some extent in any process where CO_2_ and H_2_ are present in the reaction mixture (Wang et al., 2011). As a matter of fact though, many specific papers regarding the RWGS on ceria-based catalysts have been published very recently demonstrating the increasing interest toward this topic.

#### M-CeO_2_ and CeO_2_ Based Catalysts

Most of the materials investigated contain noble (Pt, Ru) or transition metals (Ni, Cu, Co, Fe) as the active phase, but also pure (Kovacevic et al., 2016; Liu Y. et al., 2016) or doped ceria (Lin et al., 2015) has been reported to be active for the RWGS. Moreover, the addition of CeO_2_ as a dopant promotes the RWGS activity of other oxides such as Ga_2_O_3_ (Zhao et al., 2012) and In_2_O_3_ (Wang W. et al., 2016) and Zn-doped ceria (Lin et al., 2015). The activity has been ascribed to the abundance of vacancies which can favor the adsorption and the activation of CO_2_. Nevertheless the work by Kovacevic et al. suggests that there are other factors that should be considered, as for example the lattice microstrain and/or the intrinsic reactivity of different ceria facets. Any correlation of catalytic activity with the number of oxygen vacancies nor with the sample reducibility has been observed (Kovacevic et al., 2016). An indication toward the structure sensitivity of the reverse water gas shift on ceria comes from the work by Lin et al. who investigate the reactivity of copper supported on ceria nanorods and nanospheres (Lin et al., 2018). The authors explain the higher activity of Cu/CeO_2_ nanorods with an enhanced CO_2_ dissociative adsorption on CeO_2_(110) terminations which leads to the preferential formation of active bidentate carbonates and formates intermediates. In the paper by Kovacevic et al. the most reactive plane appears to be the (100) of ceria nanocubes [but they do not consider samples exposing (110) facets], so in this respect a more comprehensive study would be desirable in order to discriminate clearly the reactivity of different ceria planes for the RWGS reaction, in presence or in absence of a supported metal.

The metal-support interaction (Panaritis et al., 2018; Ronda-Lloret et al., 2018; Wang and Liu, 2018) as well as the effect of ceria in the dispersion of the active phase (Wang et al., 2013; Lu and Kawamoto, 2014; Wang L. et al., 2017; Sun et al., 2019) have a strong effect on the activity for the RWGS reaction. The role of ceria in dispersing the active phase seems particularly relevant in determining the selectivity toward CO. Different authors in fact report that large metal particles are in general selective toward methane, whereas highly dispersed nanoparticles favor the RWGS over methanation (Lu and Kawamoto, 2014; Wang L. et al., 2017; Aitbekova et al., 2018). The interesting paper by Aitbekova et al. nicely demonstrates how the selectivity toward CO with respect to methane can be tuned by a low temperature restructuring of the catalyst. In this case Ru/CeO_2_, gives rise to single-site RuO_x_ species in strong interaction with ceria. The same effect is only partially observed for Ru/Al_2_O_3_ and Ru/TiO_2_, indicating a key role of ceria in the re-dispersion of ruthenium. This aspect is very important and should be considered in future work in light of tuning the CO_2_ hydrogenation selectivity of ceria-based catalysts. The selectivity is indeed a key issue: it depends on the reaction conditions and on the nature of the metal active phase, but it has been shown that also ceria surface plays a great role in this respect, being able to activate different reaction pathways for CO_2_ leading to different products (Cheng et al., 2013; Lu et al., 2015).

#### Mechanism of Reaction

The proposed reaction mechanisms for the reverse water gas shift over ceria-based materials usually involve the adsorption and dissociation of CO_2_ on defective ceria surface followed by the formation of surface carbonate and formate species (Goguet et al., 2004; Lin et al., 2018). Surface carbonates are indicated as reaction intermediates, whereas formates are supposed to be “minor intermediates” due to their stronger bond and slower exchange rate observed in transient isotopic experiments (Goguet et al., 2004; Jacobs and Davis, 2005). However, in the recent study by Lin et al. the reported rate of decomposition of bidentate carbonates and bidentate formates is similar (Lin et al., 2018). The redox step, i.e., the formation of surface oxygen vacancies on ceria by hydrogen in presence of a metal, is necessary to initiate the reaction (particularly to activate CO_2_). This does not imply a redox mechanism for the RWGS (Wang L. C. et al., 2015; Chen et al., 2016), which is conversely supposed to follow an associative pathway (Goguet et al., 2006). All these studies, highlight that the reaction conditions adopted can affect significantly the mechanism and the reader should be aware of this when considering the number of possible pathways proposed in the literature.

### Fischer-Tropsch Reaction

#### Ceria as Promoter

The direct hydrogenation of CO_2_ to hydrocarbons, known also as CO_2_-FT, has been addressed only recently as a way for carbon dioxide upgrading. An ideal catalyst for this process should be active for both RWGS and FT synthesis, thus making its design quite complicated (Porosoff et al., 2016; Guo et al., 2018; Li and van Veen, 2018). To overcome this issue, the approach is that of designing bifunctional catalysts that couple both RWGS and FT functionalities in a single material, and a few attempts have been made also with ceria-containing formulations (Dorner et al., 2011; Samanta et al., 2017; Xie C. et al., 2017). Dorner et al. observed that the addition of ceria to a Fe/Mn/K-alumina catalyst significantly improved performances for the hydrogenation of CO_2_ to C_2_-C_5_ olefins, due to ceria promotion of the RWGS reaction. A similar approach has been described by Samanta et al. who added 5% Ce on a K/Fe-Al-O spinel observing an increase in selectivity for methane and light C_2_-C_4_ with respect to the undoped catalyst (Samanta et al., 2017). Xie et al. instead tested successfully a composite material constituted by a Pt/CeO_2_ core (RWGS functionality) surrounded by a Co-mesoporous silica shell (FT functionality) (Xie C. et al., 2017).

#### M-CeO_2_ Catalysts

Interestingly, also pure ceria has demonstrated some potentialities as a good support for direct CO_2_-FT, depending on its morphology. On Fe/CeO_2_ systems it has been reported in fact a correlation between catalytic activity and ceria morphology (Torrente-Murciano et al., 2016). The different reducibility of ceria nanoparticles, nanocubes and nanorods has been shown to change the degree of CO_2_ conversion, as well as the selectivity toward higher hydrocarbons with respect to methane and the olefin to paraffin ratio obtained. This last effect is attributed to the capability of saturating the hydrocarbon chain, higher for the more reducible support (i.e., ceria nanorods) on which paraffin's production is favored. Also the strength of CO_2_ bonding on ceria has been demonstrated to affect the selectivity of ceria-containing catalysts (Samanta et al., 2017).

### Summary and Perspective for CO_2_ Hydrogenation Catalysts Based on Ceria

Tables 2A,B summarize and provide details regarding the catalysts considered above.

**Table 2A:** Survey of Catalysts for CO_2_ hydrogenation to methanol considered in this review.

**Catalyst**	**Preparation method**	**Metal (nm)**	**T°C**	**Reaction conditions**	**XCO_**2**_ (%)**	**S_**Met**__**−OH**_(%)**	**Stability test^c^**	**References**
**CO**_**2**_ **hydrogenation to methanol on M/CeO**_**2**_ **catalysts (M = Pd, Cu, Au and bimetallic compositions)**								
4%Pd/CeO_2_	W-IMP	326	230	H_2_/CO_2_ = 3, 30 bar, W/F = 10 gcat·h/mol, mass_cat_ 0.5 g	3.1	92	nd	Tsubaki and Fujimoto, 2003
0.5%Pd/10%Cu/CeO_2_ 10%Cu/CeO_2_	CeO_2_ precipitation Cu-Pd DP	20 31	230	H_2_/CO_2_ = 3 dil., 3 MPa, W/F = 0.333 gcat·h/L	5.5 3.5	48.7 65.6	nd	Choi et al., 2017
0.5%Ca/5%Pd/5%Zn/CeO_2_	Chelating method	3–6	220	H_2_/CO_2_ = 3, 30 bar, GHSV = 2,400 mL·gcat^−1^h^−1^	7.7	100	Stable for 62 h	Malik et al., 2018
5%Cu/CeO_2_-NR	CeO_2_-NR hydrothermal W-IMP	nd	240	H_2_/CO_2_ = 3, 2 MPa, GHSV = 3 L·gcat^−1^h^−1^	~2.2	89.5	nd	Dai et al., 2017
1%Au/CeO_2_	DP	2.4	225	H_2_/CO_2_ = 9, 0.1 MPa, GHSV = 20,000 h^−1^	4.11 μmol/s·g^a^	62.2	Stable for 48 h	Vourros et al., 2017
**CO**_**2**_ **hydrogenation to methanol on M/CeO**_**2**_**/OX catalysts (M = Cu, OX = support oxide)**								
5%Cu/10%Y/50%CeO_2_/Al_2_O_3_	Co-impregnation support Cu W-IMP	nd	250	H_2_/CO_2_ = 5, ~30 bar, mass_cat_ 1 g, 100 mL/min	1.91 μmol/s·g^a^	86.3	nd	Wang et al., 2002
ZnOCu/CeZrO${}_{2}^{\text{b}}$	Reverse CP under ultrasound field	nd	200	H_2_/CO_2_ = 3 dil., GHSV = 8,800 mL·gcat^−1^h^−1^	5.7	88	nd	Bonura et al., 2011
CuZnAlCe^c^	CP	nd	250	H_2_/CO_2_ = 3 dil., 5 MPa, GHSV = 12,000 mL·gcat^−1^h^−1^	23.6	45.9	nd	Gao et al., 2013
CuZn/CeZrO${}_{2}^{\text{d}}$	CP	nd	230	H_2_/CO_2_ = 3, 3 MPa, GHSV = 12,000 mL·gcat^−1^h^−1^	22.8	53.0	nd	Ban et al., 2014
La_0.8_Ce_0.2_Cu_0.7_Zn_0.3_O_x_	Sol-gel	nd	250	H_2_/CO_2_ = 3, 5 MPa, GHSV = 3,600 h^−1^	8.1	63.3	nd	Zhan et al., 2014
30%Cu/35%CeO_2_/35%ZrO_2_	CP	nd	250	H_2_/CO_2_ = 3 dil., 3 MPa, GHSV = 7,500 mL·gcat^−1^h^−1^	14.3	53.8		Shi et al., 2018

a *CH_3_OH produced*.

b *40%CuO, 13%ZnO, 9%CeO_2_, 36%ZrO_2_ (wt%)*.

c *51%Cu, 24%Zn, 22.5Al_2_O_3_, 2.5%Ce (mol%)*.

d *54.7%CuO, 25.7%ZnO, 14%ZrO_2_, 5.6%CeO_2_ (wt%)*.

**Table 2B:** Survey of Catalysts investigated for the reverse water gas shift reaction (rWGS) considered in this review.

**Catalyst**	**Preparation method**	**Metal (nm)**	**T°C**	**Reaction conditions**	**XCO_**2**_ (%)**	**S_**CO**_ (%)**	**Stability test^c^**	**References**
**RWGS on CeO**_**2**_ **and on Ce-doped oxides**								
CeO_2_-NC CeO_2_-NR	Hydrothermal synthesis	/	560	3%H_2_, 37%CO_2_ dil., 30 mL/min	<5	80^a^ 75^a^	5% in 5 h 20%in 5 h	Kovacevic et al., 2016
CeO_2_-NC CeO_2_-NR 1%Ni/CeO_2_-NC	Hydrothermal synthesis Ni W-IMP	/	700	H_2_/CO_2_ = 1, 100 mL/min, V_cat_ = 5mL	28 23 42	100 100 100	nd	Liu Y. et al., 2016
Ga_2_O_3_-CeO_2_ (Ga:Ce = 99:1 mol)	Thermal decomposition	/	500	H_2_/CO_2_ = 1, 40 mL/min	10.99	100	nd	Zhao et al., 2012
In_2_O_3_-CeO_2_ (1:1 wt)	CP	/	500	H_2_/CO_2_ = 1, 40 mL/min, mass_cat_ = 0.05 g	20	100	nd	Wang N. et al., 2016
**RWGS on M/CeO**_**2**_ **(M = Ni, Co, Cu, Ru)**								
1%Ni/CeO_2_	CP^b^	nd	700	H_2_/CO_2_ = 1, 100 mL/min, mass_cat_ = 0.05 g	38	100	nd	Wang et al., 2013
1-5%Ni/CeO_2_	Thermal decomposition	nd-35	500	H_2_/CO_2_ = 1, 240 mL/min, mass_cat_ = 1 g	3–20	100–80	nd	Lu and Kawamoto, 2014
Ca_1_Ni_0.1_Ce_0.033_	Sol-gel combustion	12	650	Capture step: 15%CO_2_, 100mL/min Hydrogenation step: 5%H_2_, 100 mL/min	51.8	100	Stable for 20 cycles	Sun et al., 2019
1-10%Co/CeO_2_	CP	<5–>10	500	H_2_/CO_2_ = 1, GHSV = 300,000 mL·gcat^−1^h^−1^	2-31	100–88	5% in 60 h^c^	Wang L. et al., 2017
5%Co/CeO_2_	Colloidal combustion synthesis	5	600	H_2_/CO_2_ = 1, GHSV = 600,000 mL·gcat^−1^h^−1^	174.9 μmol/s·g^d^	99.8	2% in 10 h	Wang and Liu, 2018
5%Cu/CeO_2_-NR	Hydrothermal synthesis Cu W-IMP	>4	400	H_2_/CO_2_ = 5 dil., GHSV = 150,000 mL·gcat^−1^h^−1^	50	nd	nd	Lin et al., 2018
Cu-CeO_2_ (Cu:Ce = 20:80 mol)	Cu-MOF impregnated with Ce precursor^e^	nd	400	H_2_/CO_2_ = 1, 50 mL/min, mass_cat_ = 0.1 g	22	100	5% in 20 h	Ronda-Lloret et al., 2018
0.5%Ru/CeO_2_	Ru NP colloidal synthesis Deposition on CeO_2_	nd	240	H_2_/CO_2_ = 4 dil., mass_cat_ = 0.02 g	<5	98	Stable for 14 h	Aitbekova et al., 2018

a *CO produced (μmol/min·g)*.

b *In presence of NaOH and Na_2_CO_3_ (1:1)*.

c On 2%Co/CeO_2_ at 600°C and GHSV = 600,000 mL·gcat^−1^h^−1^

d *Calculated after 57 h of reaction*.

e *Followed by flash pyrolysis*.

To conclude, among the several studies regarding CO_2_ hydrogenation over ceria-based catalysts two main general issues can be identified for future tailoring and improvement of these materials. The first one is mainly related to the interaction between CeO_2_ and the supported metal, and different strategies can be found in both experimental and theoretical works to enhance the metal-ceria interplay. The second issue involves the tuning of ceria surface acid/base and redox properties, which have been found to influence strongly the pathway for CO_2_ activation (Lu et al., 2015).

**Figure 3:**
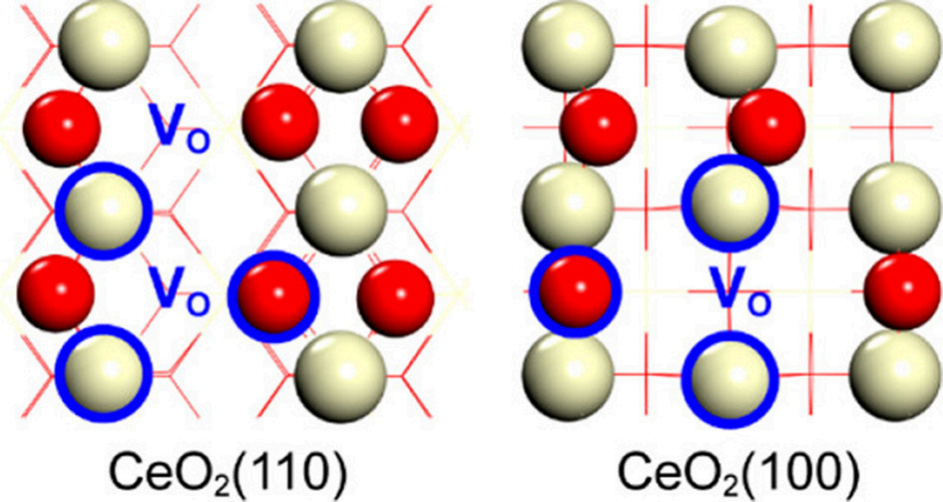
Schematic of solid FLPs on CeO_2_(110) and CeO_2_(100) constructed by surface oxygen vacancy regulation. White and red balls represent Ce and O atoms, respectively. Atoms labeled by blue circles represent the Lewis acid (Ce) or Lewis base (O) of solid FLPs. The position of oxygen vacancy is labeled by V_O_ in blue (Huang et al., 2018, Reprinted with permission from American Chemical Society).

In this respect, an interesting and very recent approach is suggested by the work of Huang and coworkers who have demonstrated the possibility of creating series of solid frustrated Lewis pair (FLPs) sites on the surface of ceria (Zhang et al., 2017; Huang et al., 2018). FLPs are constituted by Lewis acids and bases which are sterically prevented from interacting with each other (thus called “frustrated”). The concept has been firstly proposed for homogeneous hydrogenation reactions (Stephan, 2009), and has been then successfully transferred to heterogeneous catalysts for CO_2_ hydrogenation (Ghuman et al., 2015, 2016). Thanks to their unique configuration, these sites can dissociate hydrogen to produce a proton site in proximity to a hydride site that together constitute a very reactive environment for CO_2_ activation. In the case of ceria, FLPs are generated by the abundance of surface defects which can be used to prevent the formation of classical adjunct Lewis acid-base couples as shown in Figure 3.

This peculiar surface configuration of CeO_2_ has already been proven to be very active for the hydrogenation of alkenes and alkynes (Zhang et al., 2017) and can represent a very promising step forward for the exploitation and improvement of ceria-based materials for the valorization of CO_2_ through hydrogenation reactions.

## CO_2_ Reforming of Methane

Another process for the valorization of CO_2_ is its use as oxidizing agent in the process of reforming of methane (methane dry reforming, MDR). This process involves the utilization of methane and carbon dioxide, which are greenhouse gases, to produce syngas (CO and H_2_) as building block for the production of liquid fuels and chemicals (Lavoie, 2014). Since it consumes CO_2_, MDR represents an highly efficient way to reduce the carbon footprint of the world's growing consumption of methane (associated with the exploitation of natural gas) in comparison to other reforming processes.

The overall reaction (Equation 3)

(3) $${\text{CH}}_{\text{4}}\text{ + }{\text{CO}}_{\text{2}}\;=\text{2CO + }{\text{2H}}_{\text{2}}\;(\Delta {\text{H}}_{\text{298K}}\;=\text{ 247kJ/mol})$$

is thermodynamically unfavorable since implies the activation of two very stable molecules and requires temperatures higher than 1,000°C to obtain significant conversion in stoichiometric conditions (Nikoo and Amin, 2011), while a CO_2_/CH_4_ ratio higher than 3 would be necessary to have good conversion at lower temperature (750°C) (Li et al., 2008; Nikoo and Amin, 2011).

Actually, the MDR is a complex process that implies several equilibria depending on the operating temperature and can lead to the formation of dimethylether, methanol, alkanes and alkenes and carbon as side products. In the range of 650–1,000°C the main side reaction is the RWGS (reaction a) (Lavoie, 2014) along with a series of reactions (reactions b-e) that in stoichiometric conditions lead expectedly to the formation of carbon.

- CO_2_ + H_2_ ↔ CO + H_2_O          (ΔH_298K_ = 41 kJ/mol)
- CH_4_ ↔C + 2H_2_          (ΔH_298K_ = 74.9 kJ/mol)
- 2CO ↔CO_2_ + C          (ΔH_298K_ = −172 kJ/mol)
- CO_2_ + 2H_2_ ↔ C + 2H_2_O          (ΔH_298K_ = −90 kJ/mol)
- CO + H_2_↔C + H_2_O          (ΔH_298K_ = −131 kJ/mol)

Several transition metals (Ni, Ru, Rh, Pd, Ir, Pt, Co) supported on various supports have been investigated to catalyze the conversion of both CO_2_ and CH_4_ at intermediate temperatures (500–800°C), with Rh, Pt, and Ru (Pakhare and Spivey, 2014) showing high activity, thermal stability and a remarkable resistance to the carbon deposition. Despite the optimal properties of noble metals in this process, nickel would be the metal selected for an industrial development of the process because it is easily available, cheap and likewise active (Lavoie, 2014; Bian et al., 2017). However, nickel is prone to be deactivated by carbon deposits since it catalyzes the cracking process of methane while having a poor activity for the dissociation of CO_2_; moreover it can easily sinter at the MRD operating temperature. Different approaches have been followed to tackle these challenges (Li and Gong, 2014) such as alloying/doping Ni with other metals (Cu, Co, Pt, Pd, Sn, Na, K, Ca), or confining/embedding it in well-defined environments (cavities, channels, core-shell) in appropriate supports (Bian et al., 2017). Ni has been dispersed on several supports like Al_2_O_3_, ZrO_2_, TiO_2_, MgO, MgAlO, SiO_2_, and CeO_2_, or Ce_x_M_1−x_O_2_, (M = Zr, Pr, Gd) etc. (Usman et al., 2015).

Table 3 summarizes the most recent studies on dry reforming catalysts based on CeO_2_ or on its parent oxides.

**Table 3:** Survey of MDR Catalysts investigated from 2016–2018 organized according the use of CeO_2_ as support, promoter or component of a solid solution.

**Catalyst**	**Preparation method**	**Metal (nm)**	**T°C**	**Conditions of reaction**	**XCO_**2**_ (%)**	**XCH_**4**_ (%)**	**H_**2**_/CO**	**Stability testc**	**References**
**CERIA AS SUPPORT**									
3.5% Ni/CeO_2_	SF-CP, IW-IMP	–	750	CH_4_/CO_2_ = 1.5, GHSV = 8,000 h^−1^	80	48	0.93	nd	Pappacena et al., 2018
5% Ni/CeO_2_	W-IMP	8.7	500	CH_4_/CO_2_ = 1 dil., GHSV = 4.8 × 104 h^−1^	42^a^	30	0.7	stable for 10 h	Li M. et al., 2018
5%Ni-CeO_2_	CP+W-IMP of Ni	2.6	600	CH_4_/CO_2_ = 1 dil., 25 ml/min	28	60	0.93	70% in 24 h	Wolfbeisser et al., 2016
5%Ni/CeO_2_NRs	HS+IW-IMP Ni	7.8^b^	700	CH_4_/CO_2_ = 1 dil., WHSV = 36,000 mL h^−1^·gcat^−1^	77.7^b^	75.4^b^	0.97	3% in 50 h	Wang N. et al., 2016
Ni/mpCe_1−*x*_Ni_x_O_2−*y*_	CP+template	3.9–5.2	800	CH_4_/CO_2_ = 1, WHSV = 12,000 mL h^−1^·gcat^−1^	94	98	0.98	Stable in 40 h	Deng et al., 2016
Ni/nCe_1−*x*_NixO_2−*y*_	CP	3.9–5.2	800	CH_4_/CO_2_ = 1, WHSV = 12,000 mL h^−1^·gcat^−1^	76.8	84	0.96	40% in 40 h	Deng et al., 2016
5.2%Ni-SiO_2_@CeO_2_	Ni-phyllosilicate route+CP of CeO_2_	3–5	750	CH_4_/CO_2_ = 1.5, GHSV = 200 L h^−1^·gcat^−1^	0.55^d^	0.43^d^	0.88	10% in 72 h	Das et al., 2018
8.6%Ni/SiO_2_ (^*^)	Ni-phyllosilicate route	6–8	750	CH_4_/CO_2_ = 1.5, WHSV = 200 L h^−1^·gcat^−1^	0.38^d^	0.32^d^	0.93	100% in 72 h	Das et al., 2018
10%NiCe@m-SiO_2_	specific synthesis	3.2	750	CH_4_:CO_2_ = 1:1, WHSV = 6000 mL h^−1^·gcat^−1^	95.2	90	nd	stable in 40 h	Zhao et al., 2016
Ir/CeO_2_	DP	6	800	CH_4_/CO_2_ = 1, WHSW = 18,000 mL h^−1^·gcat^−1^	61	51	nd	9% in 100 h	Wang F. et al., 2017
0.86Pd/CeO_2_	SF-CP	1–15	800	CH_4_/CO_2_ = 1, WHSW = 70,000 mL h^−1^·gcat^−1^	96	93	0.8	stable for 12 h	Singha et al., 2017
10%Co/CeO_2_	IW-IMP	nd	550	CH_4_/CO_2_ = 1 dil., GHSW = nd	11	8	0.96	nd	Zhang et al., 2018
**CERIA AS PROMOTER**									
15%Ni-10%Ce/Cu-Clay	W-IMP of Ni and Ce	15.2	750	CH_4_/CO_2_ = 1 dil., GHSV = 20,000 h^−1^.	75	68	0.8	nd	Liu H. et al., 2017
14.6 wt%Ni/CeO_2_/YSZ	W-IMP	15	750	CH_4_/CO_2_ = 1, GHSV = 120,000 mL h^−1^·gcat^−1^	60	70	0.76	17%in 30 h	Muñoz et al., 2017
10%Ni/(1–12)%CeO_2_/Al_2_O_3_	W-IMP CeO_2_ on Al_2_O_3_+W-IMPNi	4.2–4.7	550	CH_4_/CO_2_ = 1, dil., WHSV = 120,000 mL h^−1^·gcat^−1^	69–58	47–57	0.63	stable in 5 h	Damyanova et al., 2018
12%Ni-5%Ce/Mg-Al-O	Mg-Al-O by CP, Ni and Ce IW-IMP	7.1	700	CH_4_/CO_2_ = 1, dil., WHSV = 72,000 mL h^−1^·gcat^−1^	67	70	0.96	stable for 20 h^e^	Fang et al., 2018
3%NiFe/6%CeO_2_-ZrO2-Al_2_O_3_	W_IMP	1–3	750	CH_4_/CO_2_ = 1, WHSV = 30,000 mL h^−1^·gcat^−1^	80	70	0.61^h^	22% in 20 h	Aw et al., 2016
3%NiW/6%CeO_2_-ZrO2-Al_2_O_3_	W_IMP	nd	750	CH_4_/CO_2_ = 1, WHSV = 30,000 mL h^−1^·gcat^−1^	35	30	0.31^h^	76% in 20 h	Aw et al., 2016
Sn_0.02_Ni/20%CeAl_2_O_3_	W-IMP CeO_2_ + W-IMP Ni and Sn	20–23	700	CH_4_/CO_2_ = 1, dil., GHSV = 30,000–120,000 mL h^−1^·gcat^−1^	90^f^	80^f^	0.9^f^	68%^f^ in 20 h	Stroud et al., 2018
Hydralcite/7%Ni3%Ce	CP	8	550	CH_4_/CO_2_ = 1 dil., GHSV = 20,000 h^−1^	37	29	0.74	stable for 5 h	Dȩbek et al., 2017
3%CoFe/6%CeO_2_-ZrO_2_-Al_2_O_3_	W_IMP	1–3	750	CH_4_/CO_2_ = 1, WHSV = 30,000 mL h^−1^ gcat^−1^	87	80	0.69 h	1.2 % in 20 h	Aw et al., 2016
3%CoW/6%CeO_2_-ZrO_2_-Al_2_O_3_	W_IMP	nd	750	CH_4_/CO_2_ = 1, WHSV = 30,000 mL h^−1^ gcat^−1^	65	61	0.35 h	59% in 20 h	Aw et al., 2016
**CERIA FORMING SOLID SOLUTIONS**									
7.9%Ni/CeO_2_–ZrO_2_	W-IMP	15.2	800	CH_4_/CO_2_ = 1.5 dil., WHSV = 120,000 mL h^−1^·gcat^−1^	86	60	0.93	30% in 28 h	Goula et al., 2017
3%Ni/Ce_0.38_ Zr_0.62_O_2−*d*_	DP	9.8	750	CH_4_/CO_2_ = 1 dil., WHSV = 67,800 mL h^−1^ gcat^−1^	88	80	0.74	40% in 20 h	Vasiliades et al., 2018
3.5% Ni/Ce_0.8_Zr_0.2_O_2_	SF-CP, IW-IMP	–	750	CH_4_/CO_2_ = 1.5, GHSW = 8000 h^−1^	93	40	0.93	nd	Pappacena et al., 2018
Ni/Ce_*x*_Zr_1−*x*_ O_2_	alcohols supercritical synthesis	nd	700	pulse experiments	0.9	100	>0.9	nd	Simonov et al., 2017
5%Ni-Ce_0.6_Zr_0.4_O_2_	CP+W-IMP of Ni	3.3	600	CH_4_/CO_2_ = 1 dil., 25 ml/min	70	52	0.63	46% in 24 h	Wolfbeisser et al., 2016
5%Ni-Ce_0.6_Zr_0.4_O_2_	SF-CP+W-IMP of Ni	0.1	600	CH_4_/CO_2_ = 1 dil., 25 ml/min	2	2	nd	nd	Wolfbeisser et al., 2016
5 wt%Ni/Ce_0.8_Zr_0.2_O_2_	SG support, W-IMP metal	14.9	750	CH_4_/CO_2_ = 1 dil., GHSV = 30,000 h^−1^	77.2	67.9	0.95	nd	Makri et al., 2015
5 wt%Ni/Ce_0.5_Zr_0.5_O_2_	SG support, W-IMP metal	15.6	750	CH_4_/CO_2_ = 1 dil., GHSV = 30,000 h^−1^	19.7	26.1	0.32	nd	Makri et al., 2015
5 wt%Ni/Ce_0.8_Pr_0.2_O_2_	SG support, W-IMP metal	27.7	750	CH_4_/CO_2_ = 1 dil., GHSV = 30,000 h^−1^	83.4	71.5	1.2	<6% in 50 h	Makri et al., 2015
5 wt% Ni/Ce_x_Pr_1−*x*_O_2−δ_ (0 ≤ x ≤ 1)	SG supports; W-IMP Ni	30–35	750	CH_4_/CO_2_ = 1 dil., GHSV = 30,000 h^−1^	62–85^g^	67–80^g^	0.75–1.5^g^	2%-16.5%^g^	Vasiliades et al., 2016
3.5% Ni/Ce_0.8_Nd_0.2_O_1.9_	SF-CP, IW-IMP	–	750	CH_4_/CO_2_ = 1.5, GHSW = 8000 h^−1^	80	48	0.81	Stable in 8 h	Pappacena et al., 2018
12%Ni/Ce_0.8_Gd_0.2_O_2_	CP	6.7	800	CH_4_/CO_2_ = 1 dil., GHSV = 28,800 h^−1^	95	92	1	stable for 100 h	Gurav et al., 2017
12%Ni/Ce_0.8_Gd_0.2_O_2_	W-IMP	16.5	800	CH_4_/CO_2_ = 1 dil., GHSV = 28,800 h^−1^	82	78	0.9	nd	Gurav et al., 2017
12%Ni/Ce_0.8_Gd_0.2_O_2_	SG	8.2	800	CH_4_/CO_2_ = 1 dil., GHSV = 28,800 h^−1^	93	88	0.95	nd	Gurav et al., 2017
3.5% Ni/Ce_0.64_Zr_0.16_Nd_0.20_O_1.9_	SF-CP, IW-IMP	–	750	CH_4_/CO_2_ = 1.5, GHSW = 8000 h^−1^	97	60	1	Stable in 8 h	Pappacena et al., 2018
3.5% Ni/Ce_0.8_Zr_0.13_Nd_0.07_O_1.96_	SF-CP, IW-IMP	–	750	CH_4_/CO_2_ = 1.5, GHSW = 8000 h^−1^	97	60	1	Stable in 8 h	Pappacena et al., 2018
1.8%Pt+Ni/PrSmCeZrO/YSZ	Pechini	nd	750	CH_4_/CO_2_ = 1 dil. WHSV = 144923 mL h^−1^ gcat^−1^	55	62	0.71	nd	Bobrova et al., 2016
1.8%Pt/PrSmCeZrO	Pechini	nd	750	CH_4_/CO_2_ = 1 dil.; WHSV = 144923 mL h^−1^ gcat^−1^	42	60	0.65	nd	Bobrova et al., 2016
0.13–0.51 %Pd-1.39 wt%Ni 1.0 wt%Mg/Ce_0.6_Zr_0.4_O_2_	CP support, IW-IMP metal	nd	450	CH_4_/CO_2_ = 1 dil., GHSV = 68,000 h^−1^	nd	1.43^i^	0.39–0.41	nd	Elsayed et al., 2016
0.15%Pd/Ce_0.6_Zr_0.4_O_2_	CP support, IW-IMP metal	nd	450	CH_4_/CO_2_ = 1 dil., GHSV = 68,000 h^−1^	nd	nd	0.24	nd	Elsayed et al., 2016
Ir/Ce_0.9_Pr_0.1_O_2_	DP	4.5	800	CH_4_/CO_2_ = 1 , WHSV = 18,000 mL h^−1^·gcat^−1^	76	61	nd	1% in 100 h	Wang F. et al., 2017
Ir/Ce_0.9_Zr_0.1_O_2_	DP	3.5	800	CH_4_/CO_2_ = 1, WHSV = 18,000 mL h^−1^·gcat^−1^	73	57	nd	1.5% in 100 h	Wang F. et al., 2017
Ce_0.8_Pr_0.2_O_2−δ_	SG	–	750	CH_4_/CO_2_ = 1 dil., GHSV = 30,000 h^−1^	0.2	0.1	nd	nd	Vasiliades et al., 2016
Ce_0.8_Pr_0.2_O_2−δ_	SG	–	750	CH_4_/CO_2_ = 1 dil., GHSV = 30,000 h^−1^	0.2	0.1	nd	nd	Makri et al., 2015

*CP = coprecipitation; W-IMP = wet impregnation; IW-IMP = incipient wet impregnation; SF-CP = surfactant assisted co-precipitation; HS = hydrothermal synthesis; SG = sol gel synthesis NRs = nanorods; DP = deposition precipitation; dil. = diluted*;

a after 5 h on the stream;

b for nanorods, NR;

c decrease in % of methane conversion;

d mol conv/min^*^gNi;

e tested at 800°C;

f at GHSW = 60,000 ml/h gcat;

g 20–80 mol% Pr;

h after 25 h;

i Rate (mol/hr/g.cat)^*^102, (^*^) introduced for comparison.

### CeO_2_ as Catalyst

The activity of CeO_2_ alone (BET SA: 23 m^2^/g) in the dry reforming process was investigated by Laosiripojana and Assbumrungrat (2005). Results were benchmarked with those of a typical Ni/Al_2_O_3_ catalyst._._ Despite a relative low conversion (32% for CH_4_ at 900°C) ceria proved to be a catalyst much more stable, showing a deactivation of 7% over time in comparison to that underwent by Ni/Al_2_O_3_ (96%). In this study, which is one of the few that has investigated the dry reforming activity of purer ceria (Otsuka et al., 1997), it is also pointed out that the main drawback of ceria is its basic nature that would favor the adsorption and hydrogenation of CO_2_ at disadvantage of methane activation.

### Ni-CeO_2_ Catalysts

**Figure 4:**
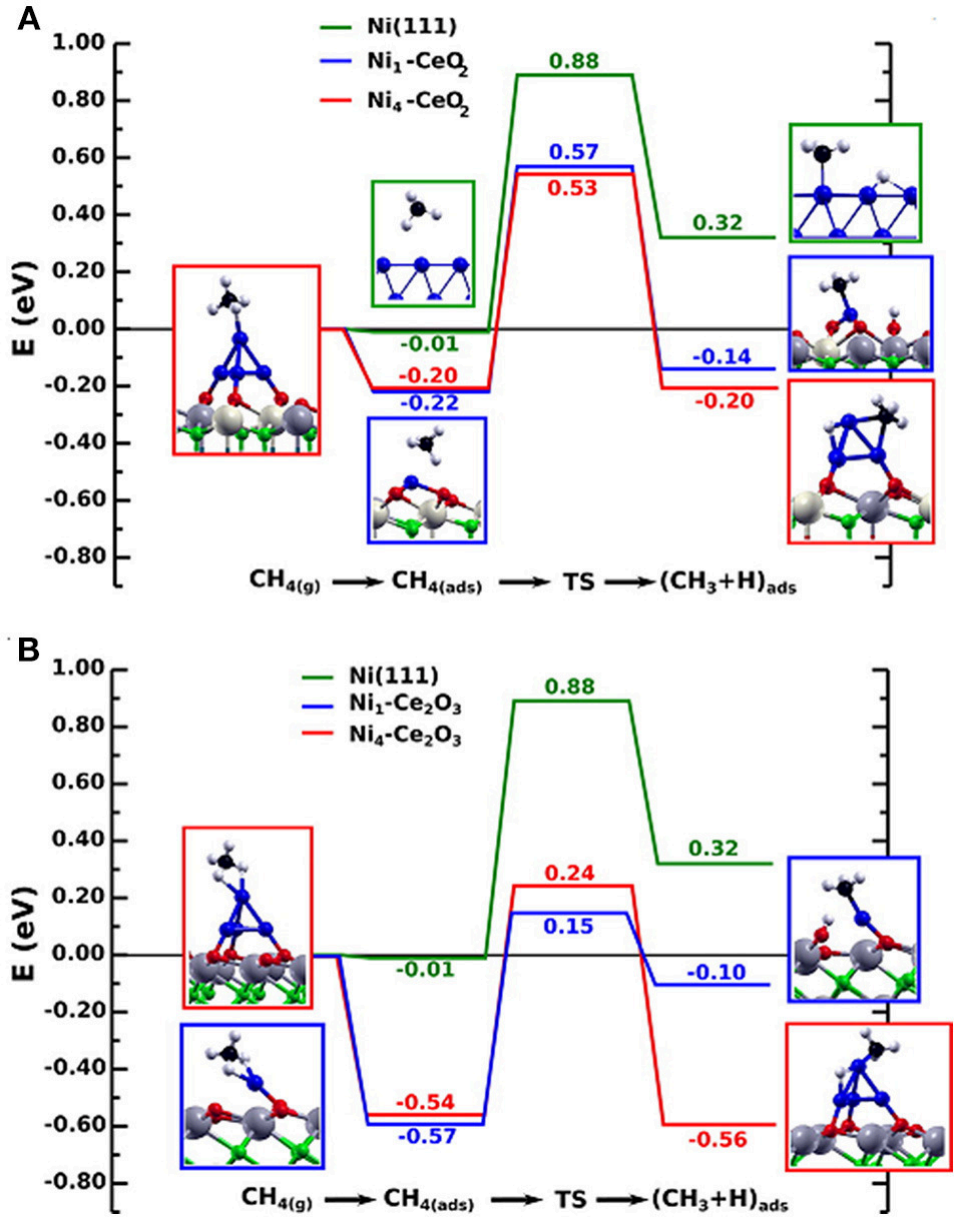
Reaction energy profile for the CH_4_ → CH_3_ + H reaction on isolated Ni atoms and Ni_4_ clusters on the CeO_2_(111) **(A)** and Ce_2_O_3_(0001) surfaces **(B)**, in comparison to Ni(111). The structures shown to the left and right of the reaction pathways correspond to the side views of the optimized molecularly adsorbed and dissociated states used in the search of the transition state structure. All energies are relative to CH_4_ in the gas phase. Reproduced from Lustemberg et al. (2016), with permission of ACS publications.

From Table 3 it is clear that in the dry reforming process ceria has been used as support or promoter; the metal component can be a noble metal but very often Ni, a Ni alloy or Co. Therefore, it is evident that CeO_2_ has a crucial role in the development of durable nickel dry reforming catalysts operating at intermediate temperature. CeO_2_ strongly interacts with Ni and recently experimental and DFT calculation demonstrated that the strong metal-support interaction between Ni and CeO_2_ can activate and modify the electronic and chemical properties of the metal allowing the activation of methane even at room temperature (Liu Z. et al., 2016). Ambient-pressure X-ray Photoelectron Spectroscopy (XPS) analysis and Scanning Tunneling Microscopy, (STM) characterization of NiO supported on CeO_2_(111) substrate revealed that when Ni atoms or small clusters are in tight contact with ceria the methane can be activated at room temperature and the dry reforming reaction become possible at a much lower temperature than that typically reported for conventional Ni based catalysts (427° C). In this case the active phase contained small particles of metallic Ni dispersed on partially reduced ceria, and the strong metal-support interaction caused large electronic perturbations responsible of a 80% lower activation barrier for the C-H bond with respect to that of Ni (111) surface (Figure 4).

#### Ni-CeO_2_: Mechanism of Reaction

**Figure 5:**
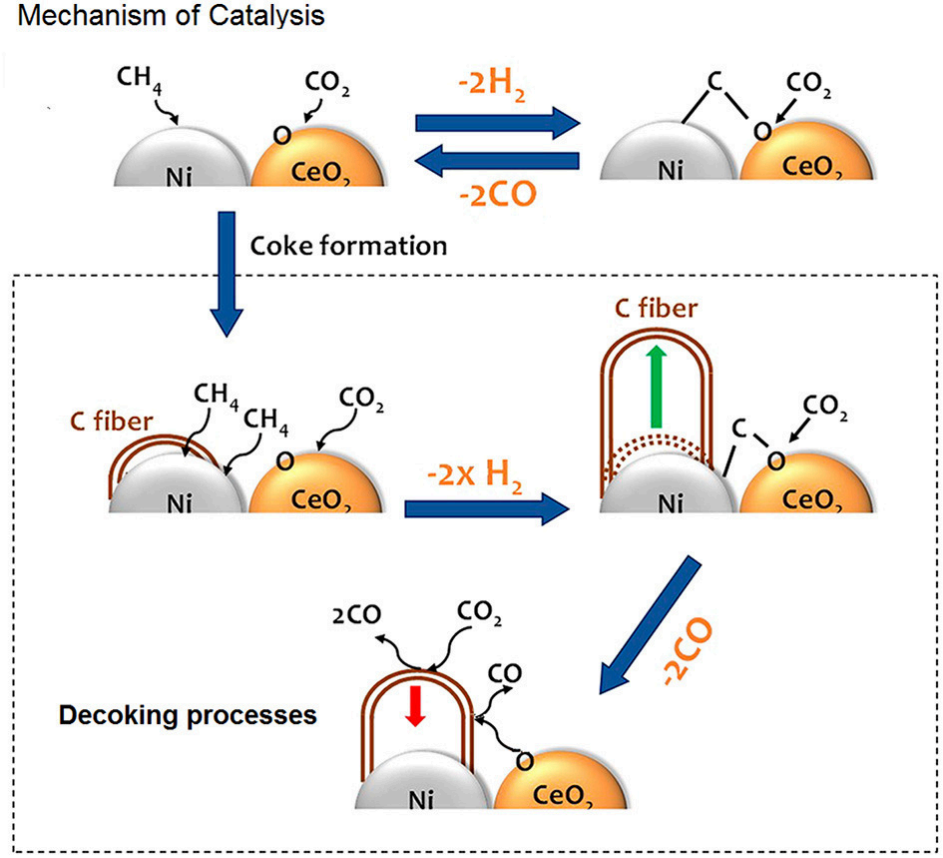
In presence of ceria the kinetics follows a Mars-van Krevelen mechanism, where active carbon is oxidized by CeO_2_, while oxygen vacancies are replenished by O from CO_2_ dissociation; the carbon fibers growth is hindered by CeO_2_, which provides extra oxygen for their gasification. Adapted from Liang et al. (2018) with permission of ACS publications.

Ni and O atoms work in cooperative way to dissociate CH_4_ molecules and once the first C-H bond of methane has been broken the sequential decomposition of the other CH_x_ intermediates into C is hypothesized to be very fast. C reacts with O present on the surface of catalyst and the resulting oxygen vacancies on the support enhance the ability of the system to adsorb and dissociate CO_2_. Figure 5 shows a cartoon with the mechanism of reactions.

This type of mechanism, which involves separate steps for the activation of methane on the metal (the rate determining step) and for the activation of CO_2_ on the support and at the interfacial sites, was suggested to be operative also in other ceria based systems (Bobin et al., 2013; Makri et al., 2015; Simonov et al., 2017).

In such systems the kinetics balance between the two steps was demonstrated to be an effective method to contrast the catalyst deactivation due to the growth of graphitic carbon filaments. Thanks to their oxygen exchange properties ceria based supports may easily provide extra oxygen incrementing the gasification rate of carbonaceous deposits.

#### Ni-CeO_2_ Catalysts: Design Strategies

**Figure 6:**
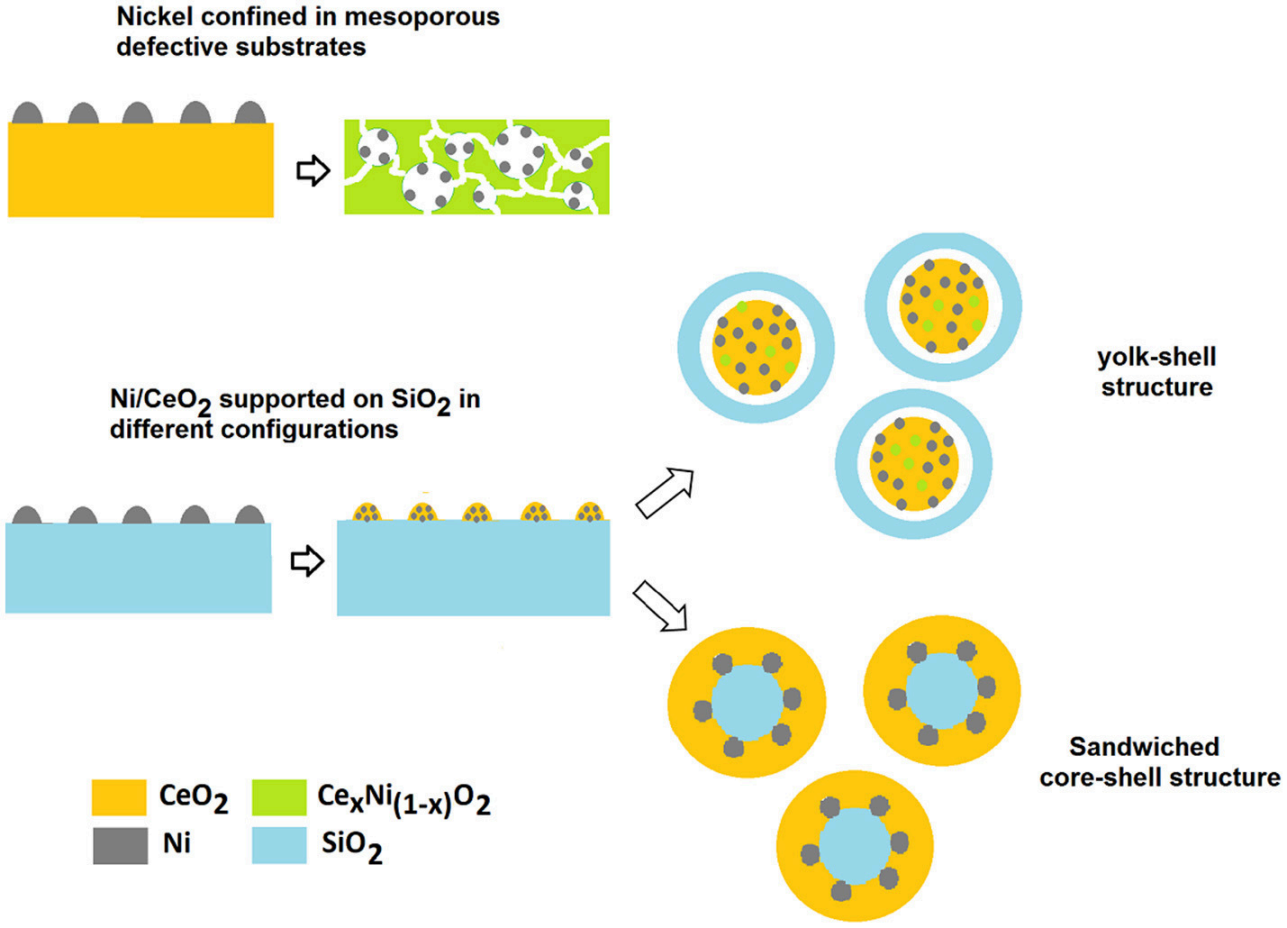
Strategies to stabilize and activate Ni/CeO_2_ catalysts.

In order to facilitate the occurrence of a support- mediated redox bi-functional mechanism for MDR a winning strategy is to improve both the dispersion of Ni and the oxygen storage capability of the support by increasing the concentration and mobility of oxygen vacancies on its surface. Figure 6 summarizes some of the strategies described below.

Deng et al. demonstrated that this can be obtained by tuning simultaneously the compositions and the morphology of the support (Deng et al., 2016). An outstanding improvement in the catalytic performance and durability of Ni nanoparticles was observed when Ni was dispersed on a solid solution of Ce_x_Ni_1−x_O_2−δ_ with a well-defined architecture.

In this system the presence of defects favors the reducibility and dispersion of Ni improving its dry-reforming activity, while the mesostructure in which Ni particles are confined works synergistically to boost the metal/support interaction. This study is a good example of the potentiality and versatility of fluorite-type ceria structure for engineering powerful MDR catalysts.

The importance of a confinement of Ni particles and of a redox bifunctional mechanism in enhancing the stability of Ni dry reforming catalysts has been also highlighted in the recent study of Das et al. (2018). In this case Ni particles are maintained well-dispersed by their encapsulation into a layer of SiO_2_, with CeO_2_ forming a sandwiched core-shell structure Ni-SiO_2_@CeO_2_. This configuration induces a change in the reaction mechanism from a mono functional pathway on the metal to a bifunctional route in which ceria has an active role in the activation of CO_2_ and in the removal of coke. The interaction between the SiO_2_ and CeO_2_ layers is pivotal for an enhanced decoking action of cerium as it contributes to an increase in the number of defects (Ce^3+^, oxygen vacancies) and thus in a greater availability of mobile oxygen on the surface of the cerium layer. The importance of optimizing the nature of CeO_2_/SiO_2_ interface has been pointed out in other reports, and the strategies adoptable for the purpose can be different due to the versatility in manufacturing SiO_2_ based nanostructured substrates (Taufiq-Yap et al., 2013; Zhao et al., 2016). It is interesting to observe that Zhao et al. obtained outstanding methane conversion of 90% at 750°C for a NiCe@m-SiO_2_ yolk-shell configuration where a SiO_2_ shell contains NiO/CeO_2_ particles. The catalyst showed similar characteristics to those of the sandwiched configuration with well confined Ni particles and the presence of CeO_2_/SiO_2_ interfaces with a high oxygen mobility. We could assume that in both configurations there is a synergic action among the three components and the confinement of Ni has a key role in influencing the properties of the mutual interfaces.

Considering the several studies published on Ni-Ce systems, it is possible to conclude that the redox properties of CeO_2_ has two mutually dependent beneficial effects. The easy reducibility of CeO_2_ promotes the reduction of nickel phases through a spillover mechanism. Additionally, the oxygen vacancies created in the ceria lattice are able to stabilize the supported metal particles; this builds a synergic system where the oxygen exchange properties of CeO_2_ are involved in a complex series of pathways to produce CO and H_2_ and inhibiting coke formation.

### Other M-CeO_2_ Catalysts

A similar synergism has been shown also in Co-Ce and Pd-Ce dry reforming catalysts (Singha et al., 2017; Zhang et al., 2018). In these systems the metal-support interaction is even stronger activating the dry reforming of methane already at 520 and 350°C, respectively. In all these catalysts the formation of a specific metal-ceria interface has a key role in decreasing the oxygen vacancy formation energy by up to a few eVs and in changing the electronic state of metal clusters (Liu et al., 2017; Ruiz Puigdollers et al., 2017).

Taking into account the crucial role of interface sites in improving catalytic activity and stability of ceria supported catalysts, several approaches have been followed to maximize their number and optimize their configurations. The properties of the metal-ceria interfaces can be tuned through thermal and redox treatments and by selecting synthesis methods aimed at reducing the size of crystallites in both the support and the metal. Another factor to be considered is the choice of the appropriate metal loading (Djinović et al., 2015). As a rule of thumb to avoid sintering phenomena and to maximize dispersion the metal loading should be in the range of 3–10% and the temperature of catalysts calcination below 500°C (Aw et al., 2014).

Low temperatures of calcination limit the sintering process especially in the case of the use of Co, probably because of the low melting point of its precursor (Co_3_O_4_, m.p., 895°C). It was reported that Co/CeO_2_ catalysts prepared via impregnation showed a 18% drop in the hydrogen yield if calcined at 600°C instead of 500°C (Abasaeed et al., 2015).

### M-Nanostructured Ceria Catalysts

Recently a plasma treatment of CeO_2_ powder before of and after its impregnation with Ni precursors has been adopted instead of a more conventional thermal treatment (Odedairo et al., 2013). The unusual treatment led to obtain (i) a clean metal–support interface, (ii) the exposure of specific surface crystallographic planes-[(111) and (100)]-for ceria, (iii) Ni clusters of 10–30 nm well-dispersed on the support surface. All these characteristics resulted in a more active and stable catalyst and would suggest that the use of nanostructured ceria support (cube, rods) may have a positive role in the dry reforming process. The study of Wang N. et al. (2016) confirms this hypothesis for Ni/CeO_2_ catalysts. Nanostructured catalysts showed the following order of reactivity Ni/Ce-nanorods>Ni/Ce-nanoctas>Ni/Ce-nanocubes>Ni/Ce-nanoparticles when tested in methane dry reforming reaction in the range of 550–750°C. The higher activity and carbon resistance of catalysts supported over CeO_2_ nanorods has been attributed to the incorporation of Ni into the ceria in a distinct configuration different from those of the other nanostructures. Ni resulted strongly anchored to the Ce-nanorods with a site geometry and coordination environment that favor the mobility of oxygen and the catalytic activity. Despite these encouraging results, the use of nanostructured ceria in MDR reaction would be limited to temperature up 700–750°C, because of their low thermal stability.

Redox and/or thermal pre-treatments can induce morphological and structural changes in the interface, strengthening the metal-support interaction or inducing inhibitory processes such as partial encapsulation of metal particles by means of the ceria support. A strong bonding between Ni and CeO_2_ that inhibited Ni particle sintering was obtained via a pre-reduction of Ni/CeO_2_ in H_2_ in the temperature range of 500–700°C, while higher temperatures of reduction (≥600°C) induced decoration/encapsulation of Ni nanoparticles by a thin layer of reduced ceria support with partial coverage of Ni surface. This modification decreased catalytic activity of Ni, nevertheless it improved its resistance to coking (Li and van Veen, 2018).

Syntheses via co-precipitation or via sol gel generally result in more active and stable catalysts with respect to those obtained via impregnation, since these approaches permit to achieve a greater Ni-ceria interaction in the final system.

### Ceria as Promoter

The method of synthesis and the ratio among the components become important when CeO_2_ is used as promoter or modifier, as in Ni/CeO_2_/Al_2_O_3_ catalysts. When added to Al_2_O_3_, CeO_2_ has beneficial impact in the dispersion of Ni and in avoiding the formation of undesired Ni Aluminate phases. Moreover, it leads to an improvement in the durability of the catalyst generally proportional to the ceria concentration (Chen et al., 2013). With regard to the impact on the activity and selectivity, it is crucial to optimize the composition in order to maximize the surface area of the metal and the interfacial surface between CeO_2_ and Ni (Damyanova et al., 2018).

The observation that ceria can change the properties of the supports and influence their interaction with NiO having a beneficial effect on the endurance of the catalysts has been reported for other systems based on MgO (Fang et al., 2018), ZrO_2_, (Kumar et al., 2008), SiO_2_, (Taufiq-Yap et al., 2013), and hydrotalcite (Debek et al., 2015). Despite the general improvement on the longevity, the effect of ceria addition on the activity and selectivity of these catalysts depends on the type of the support and can be also slightly negative. Considering the literature, it is clear that in multicomponent-catalysts the optimal composition is one in which the surface area of the metal, the basicity of the carrier oxide and the availability of surface oxygen vacancies are balanced to close the catalytic cycles involved in the dry reforming process.

### M-Doped CeO_2_ Catalysts

The same perspective of optimizing a winning composition for the process has driven the studies on supports based on ceria doped with zirconium or with rare earths elements (Gd, Sm, Pr) (Makri et al., 2015; Vasiliades et al., 2016; Wolfbeisser et al., 2016; Gurav et al., 2017; Wang F. et al., 2017).

The use of ceria-zirconia mixed oxide in the development of Ni catalysts generally leads to an improvement in the catalyst activity and selectivity; however the impact depends on the composition and on the synthesis methods, and about these aspects there is no always agreement in the literature. The controversial results sometimes reported for similar compositions are mainly related to the different approaches adopted for their synthesis. It was highlighted for example that the addition of surfactants, which are used to increase the mesoporosity and surface area of Ni/Ce_x_Zr_1−x_O_2_, can be beneficial or not, depending on the selected composition and on the type and amount of surfactant. A large amount of surfactant leads generally to an irreversible encapsulation of Ni during thermal/redox treatments (Wolfbeisser et al., 2016; Pappacena et al., 2018). Although the preparation method is an important way to develop the properties of these substrates, it should not be overlooked that the operating conditions, thermal and redox treatments often induce nanostructural and compositional changes in these oxides, which lead to materials with different surface properties and compositions from those expected. For example, Djinovic et al. demonstrated that the activity of NiCo catalysts supported by Ce_0.8_Zr_0.2_O_2_ was changing over time showing a self-activation due to the completion of the Ni particle reduction and the recrystallization of the support from a cubic to a more polyhedral shape of crystallites (Djinović et al., 2014). Therefore, in order to identify the most promising support composition for the process, efforts must be directed primarily to the understanding and control of the surface transformations of these systems under operating conditions.

The performance improvement of these materials has been correlated with the enhancement of both their OSC and their thermal resistance induced by the doping. The high thermal resistance of ceria-rich ceria-zirconia solid solutions allows a limited growth of their crystallites during thermal treatments, that in turn hinders the aggregation of Ni particles favoring metal dispersion (Kambolis et al., 2010).

Very recently, by using transient isotopic studies, a Mars-van Krevelen mechanism has been proposed to correlate the redox behavior of ceria doped support with the ability to get rid of the carbon accumulated on the metal during the dry reforming reaction. In this mechanism the support provides a pool of surface and subsurface oxygen able to remove the carbon species formed both via methane cracking and via Boudouard reaction. It has also been noted that the coke resistance of Ni catalysts on these supports is not clearly correlated with their bulk OSC properties (Makri et al., 2015). It is instead more related to their surface reducibility and to the oxygen mobility to the Ni interfacial sites. According to the literature, the advantages in the durability of MRD Ni based catalysts supported on Ce-Zr oxides are associated to ceria molar contents higher than 50%, and they are normally attributed to the crystalline cubic structure characteristic of these cerium-rich compositions (Kumar et al., 2007, 2008; Zeng et al., 2013; Nahar and Dupont, 2014; Djinović et al., 2015; Muñoz et al., 2017).

**Figure 7:**
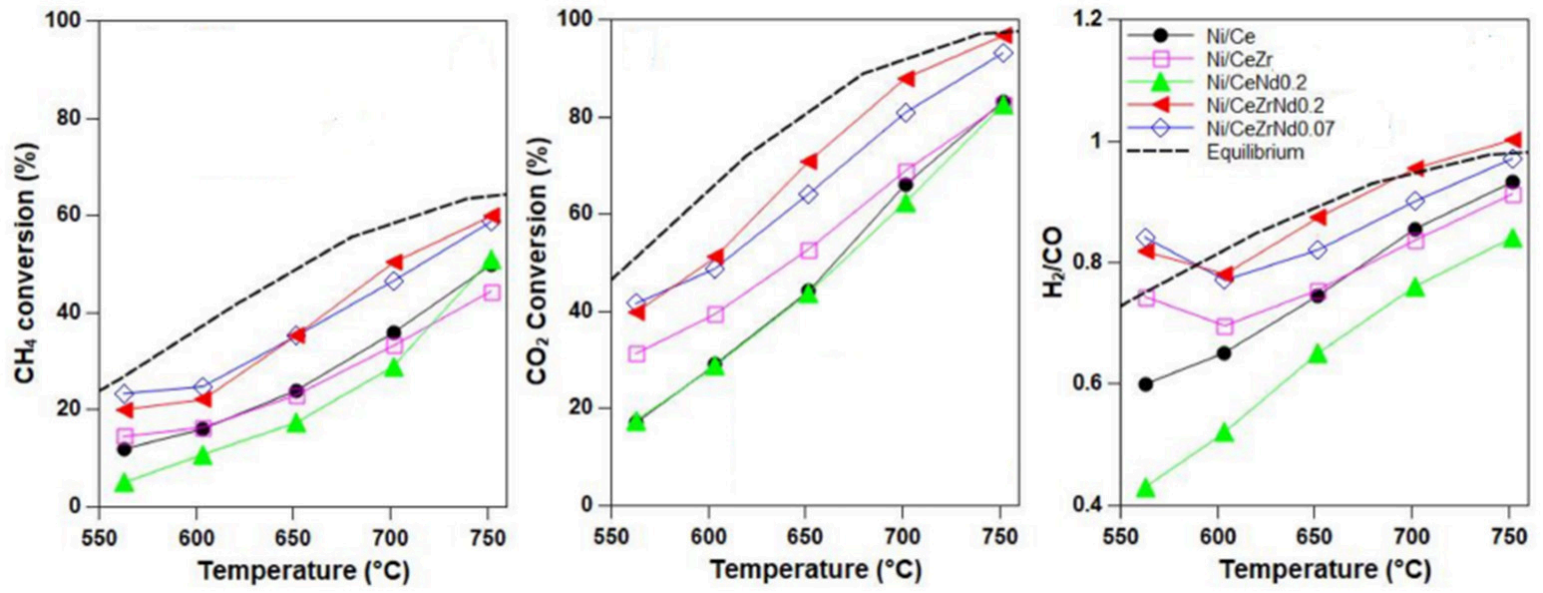
Methane dry reforming activity of Ni catalysts prepared by wet impregnation of supports of CeO_2_, CeO_2_ doped with Nd or Zr and of CeO_2_ co-doped with Zr and Nd. (GHSW = 8,000 h^−1^, CH_4_/CO = 1.5). It was possible to obtain an improvement of ceria MDR activity only by co-doping, which affects both redox and acic-basic properties of support. Adapted from Pappacena et al. (2018), “Open Access” and licensed by the respective authors in accordance with the Creative Commons Attribution (CC-BY) license.

Another aspect to be considered is that the insertion of zirconia into the ceria lattice affects its basic properties. CO_2_-TPD studies on Ni/CeZr catalysts proved that the addition of zirconium increments the number of basic sites of medium and weak strength that are crucial to enhance the conversion of methane and to favor the adsorption/activation of CO_2_ (Zeng et al., 2013; Pappacena et al., 2018). Conversely, the insertion of rare earth elements would increase the number of strong basic sites. The presence of these type of sites has been correlated with the accumulation of carbonates, that were proved to work as spectators in the ceria-zirconia solid solutions and as intermediates in rare earth doped ceria supports (Makri et al., 2015). However, the role of carbonates is still to be well elucidated in these materials. Taking into account that zirconium decreases the basicity of ceria, while rare-earth elements increase it, one can hypothesize that a correct balance between acidic and basic sites into this oxide can be achieved by a co-doping approach. Recently we have demonstrated that the co-doping of ceria with zirconium and neodymium enhanced the activity and selectivity of Ni catalysts in comparison to those singly doped (see Figure 7; Pappacena et al., 2018).

Bobrova et al. reported high performance for a Ni+Pt catalyst supported on a ceria zirconia composition co-doped with Pr and Sm (Bobrova et al., 2016). All these multicomponent systems gave H_2_/CO ratio close to 1, thus suggesting that playing with the compositions of metal phase and/or of the support can be a valid strategy to limit the activity of ceria toward the Reverse Water Gas Shift reaction (RWGS, CO_2_ + H_2_↔CO + H_2_O), which is the main issue to be addressed when ceria enters in the formulations of catalysts for the methane dry reforming process.

## Conclusions

**Scheme 1:**
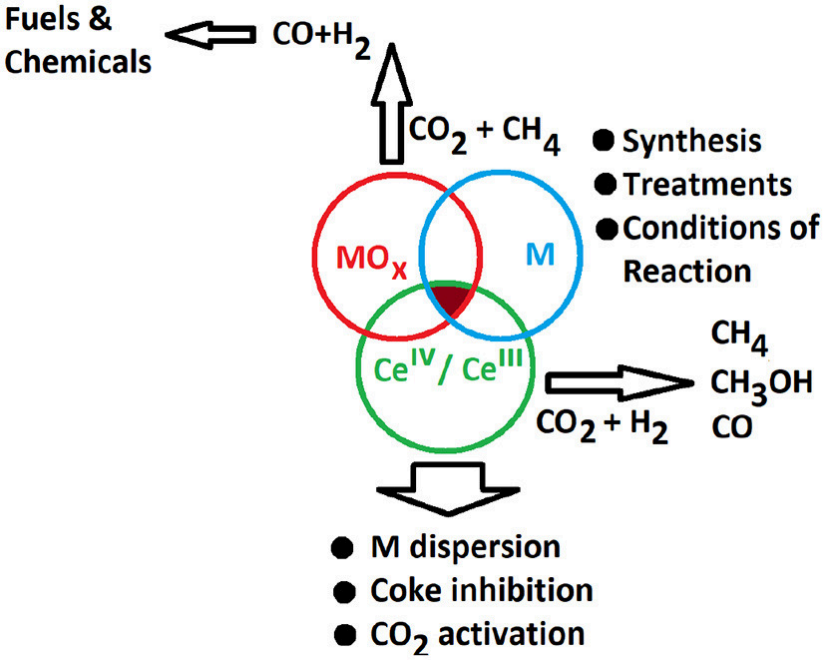
Summary of concepts reviewed in this article.

In the framework of the development of a carbon free footprint circular economy based on the valorization of CO_2_ we have reviewed the role of ceria in the development of catalysts for processes of growing relevance such as the dry reforming of methane and CO_2_ hydrogenation to CH_4_, CO and methanol. The engineering of these catalysts formulations is aimed at improving the synergic interaction between the different components (metal, support, and promoters) in order to maximize yield and selectivity (see Scheme 1).

We have shown that cerium dioxide and ceria-based materials can effectively contribute to enhance the catalytic activity through the following actions:

- **by increasing the number of active sites and their activity** CeO_2_ reducibility contributes to establish a strong metal-support interaction which promotes the mutual reducibility of the metal and the support through a spillover mechanism of H_2_. The presence of surface defects (Ce^3+^ and oxygen vacancies) allows a good anchoring of metal particles on ceria support, thus leading to a higher metal dispersion. This results in a greater number of active sites (especially in case of non-noble transition metals such as Ni, Co, Cu), which for CO_2_ reforming processes are mainly located at the metal-support interface.
- **by enhancing the selectivity and durability of catalysts**
  Metal nanoparticles and a strong metal-support interaction are the basis of electronic perturbations at the metal surface and of spillover mechanisms that drive the selectivity of processes as well as the durability of the catalyst against inhibitory reactions, such as the formation of coke. Moreover, a strong metal-support interaction may inhibit sintering processes.
-**by introducing different functionalities**
  The surface of nanostructured cerium oxide is characterized, in particular in reducing atmosphere, by the presence of oxygen vacancies V_0_ and reduced cations Ce^3+^ that, in addition to being active sites for the activation of CO_2_ in the processes, can act as acid-base pairs in the hydrogenation reactions.
-**by offering versatility of approaches in designing and engineering appropriate interfaces**
  The morphological and structural characteristics as well as acidity, basicity, redox centers of ceria and ceria based oxide depends on the method of synthesis and on the extent of doping. Moreover, these supports are highly sensible to the type of environment. This expands the possibility of optimizing surface and interface properties of catalysts. However, more in-depth studies on the behavior and the evolving of these systems under reaction conditions are needed. A better understanding of structural and compositional changes, and of the corresponding redox properties undergone by systems under operating conditions would allow the creation of intelligent interfaces capable of exploiting the chemical-physical properties of cerium-based support for, *in situ*, functional rearrangements or rejuvenation processes.

Incidentally, this work summarizes the peculiar characteristics of Ni/CeO_2_ and Ni/Ce_x_Z_r1−x_O_2_ systems since these catalysts are among the most studied catalysts in methanation and in the methane dry reforming processes. Recent studies have demonstrated that these compositions are also active in the direct conversion of methane to alcohols (Okolie et al., 2017; Lustemberg et al., 2018). Even if further studies will be necessary to understand the catalytic behavior of Ni/Ce systems under working condition and to improve their stability, it is expected that these compositions will have a significant impact in the industrial development of catalytic processes for the valorization of both CO_2_ and CH_4_.

## Author Contributions

MB wrote introduction, conclusions, and the paragraph on methane dry-reforming. SC wrote the paragraph on CO_2_ hydrogenation. AT reviewed the whole manuscript.

### Conflict of Interest Statement

The authors declare that the research was conducted in the absence of any commercial or financial relationships that could be construed as a potential conflict of interest.
